# Colonization routes uncovered in a widely introduced Mediterranean gecko, *Tarentola mauritanica*

**DOI:** 10.1038/s41598-023-43704-8

**Published:** 2023-10-04

**Authors:** Catarina Rato, Gregory Deso, Julien Renet, Michel Jean Delaugerre, Valéria Marques, Gabriel Mochales-Riaño

**Affiliations:** 1 CIBIO – Research Centre in Biodiversity and Genetic Resources, Universidade do Porto, Campus de Vairão, Rua Padre Armando Quintas 7, 4485-661 Vila do Conde, Portugal; 2 BIOPOLIS Program in Genomics, Biodiversity and Land Planning, CIBIO, Campus de Vairão, 4485-661 Vairão, Portugal; 3 Ahpam (Association herpétologique de Provence Alpes Méditerranée), Maison des Associations 384 Route de Caderousse, 84100 Orange, France; 4 Fauna Studium, Scientific Consulting, 04290 Salignac, France; 5 Conservatoire du littoral. Résidence Saint Marc, Rue du Juge Falcone, 20200 Bastia, France; 6 Institut de Biologia Evolutiva (CSIC-UPF), Passeig de la Barceloneta 37-49, 08003 Barcelona, Spain

**Keywords:** Evolution, Genetics, Molecular biology

## Abstract

In this study, we aimed to understand the contemporary and ancient colonization routes of the Moorish gecko, *Tarentola mauritanica*, using simple sequence repeats. By analyzing the genetic diversity of populations in different regions, we found that Morocco is the genetic diversity hotspot for the species, followed by the Iberian Peninsula. However, historical gene flow estimates identified the Iberian Peninsula, not Morocco, as the primary contributor of colonizing individuals, along with continental Italy to a lesser extent. Currently, mainland Italy is the main source of introduced individuals, likely due to the plant nursery trade. The study suggests that human-facilitated introductions from various geographical origins, with numerous regions colonized through continental Italy during two distinct periods, are responsible for the recurrent entry of individuals belonging to the European lineage of *T. mauritanica* into the Mediterranean and Macaronesia. These findings can inform better monitoring surveys and conservation programs by identifying putative current colonization routes of alien species.

## Introduction

It is widely known that the introduction of species into new localities may have considerable ecological and evolutionary consequences for the native species and host ecological communities^1,2^. Moreover, human globalization is leading to an increase in the rate of species’ translocation to areas outside their native geographical range^3^. Curiously, reptiles seem to be particularly prone to biological introductions, either by being one of the most often introduced animal groups, or by being notably sensitive to the impacts of alien species^4^.

Therefore, determining the routes of introduction—the geographic pathways of the propagules between the source and the introduced populations—provides not only information about the history of the colonization process, but also an understanding of the origin and construction of the genetic composition of the newly established populations^5^. Ultimately, this knowledge can have conservation implications by aiding in the design of monitoring and inspection programs for particular colonization routes or expected propagule size^6,7^.

Unfortunately, we are many times faced with very low genetic diversity and shallow divergence patterns when studying introduced populations originated from either human introductions or very recent natural colonization events, precluding the assessment of the ultimate geographic origin of the source e.g.,^8–10^. Hence, the need to use faster evolving genomic regions such as simple sequence repeats (SSRs) or commonly referred to as microsatellites. Extensive genotyping endeavours come with a high price tag and yield reduced benefits when conducting typical genetic analyses^11–13^. Consequently, research teams frequently resort to analysing a more limited set of genetic markers. Research conducted in various species, including fruit flies^11^, fish^14,15^, birds^16^, amphibians^17^, wild boars^18^, felids^19^, and beetles^20^, has shown that using a set of 6–14 microsatellite loci provides sufficient information to detect even subtle population structure.

**Figure 1:**
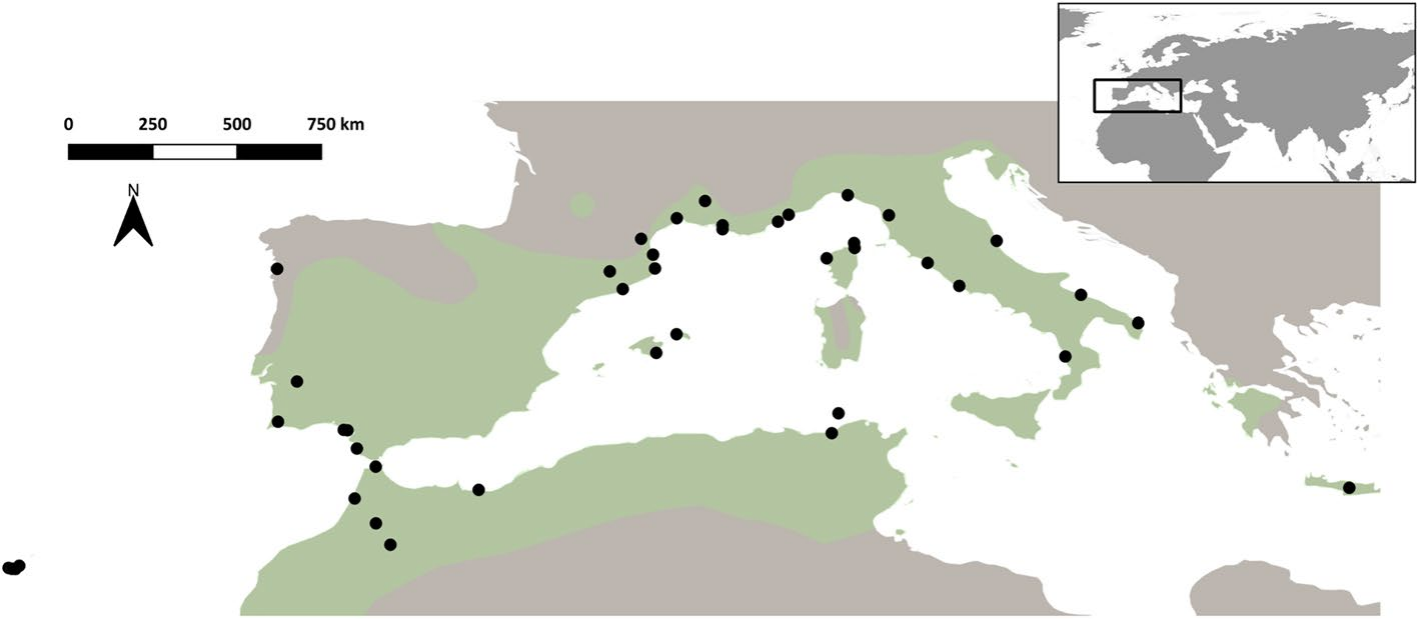
Black dots denote the location of all 44 populations of *T. mauritanica* used in this study. The native geographic distribution of the Moorish gecko modified from^21^, is represented in green. Map generated using QGIS software^95^.

The Moorish gecko, *Tarentola mauritanica* (Linnaeus, 1758) has a widespread geographic distribution across Southern Europe, the Maghreb region of North Africa, namely Morocco, Algeria and Tunisia, some Macaronesian islands, and is also present in several localities of the American continent^21^ (Fig. 1). Because this species is frequently associated with humanized infrastructures, accidental introductions into new areas have been reported. For instance, many of the populations of the Northern Mediterranean are likely to have been introduced during the Pleistocene e.g.,^22–27^, contrary to some insular populations from the Mediterranean e.g.,^28–33^ and Macaronesia e.g.,^34,35^, and the ones from the New World e.g.,^36–41^, which result from recent introductions.

The first phylogeographical studies performed on the Moorish gecko detected an extremely high mitochondrial DNA genetic variability, identifying six distinct lineages^22–26,42^, which were later recognized as putative candidate species based on a multilocus species tree study^43^. Moreover, this elevated genetic diversity is particularly evident in North Africa, with Morocco harbouring two endemic lineages and sharing most of the remaining ones with the Iberian Peninsula^23–25^. Also, the Iberian Peninsula has an endemic mitochondrial lineage^22^. Most of these mtDNA lineages have very restricted geographic ranges with considerable genetic diversity, contrasting with the pattern of the European lineage^25,26^; this clade is spread across the entire Mediterranean Basin, with all introduced populations comprised exclusively of individuals from this clade with a practically null mtDNA genetic diversity, preventing the assessment of potential gene flow routes within this group. However, the recent study from Belluardo, et al.^44^ uncovered 13 new 16S mitochondrial haplotypes within the Italian populations of the European clade, suggesting this region as a center of genetic diversity for this lineage. Moreover, their results on microsatellite data propose an overall shallow population genetic structure.

Hence, this study aims to uncover the contemporary and ancient colonization/introduction routes among several populations of the Moorish gecko belonging to the European mitochondrial clade. In order to achieve that, a battery of 11 microsatellites especially designed for this species was genotyped for 44 sampling sites distributed across the Mediterranean and Macaronesia regions. The results obtained here will ultimately explain the extant geographic range and genetic diversity composition and distribution within this widespread lineage of *Tarentola mauritanica*.

## Results

Out of the 11 genotyped microsatellite markers, three of them were removed from further analyses (Mt3, Mt14 and Mt29; but see Table S2). Both Mt3 and Mt14 were identified by MICRO-CHECKER as containing null alleles. According to GENEPOP none of the loci were in LD but most of them were not in HWE, since almost every population was also not in HWE (independently of its effective size). Therefore, we discarded the loci with the lowest *p* values and higher number of populations in disequilibrium (Mt3, Mt14 and Mt29), ending up with a total of 8 microsatellite loci for further analyses.

**Figure 2:**
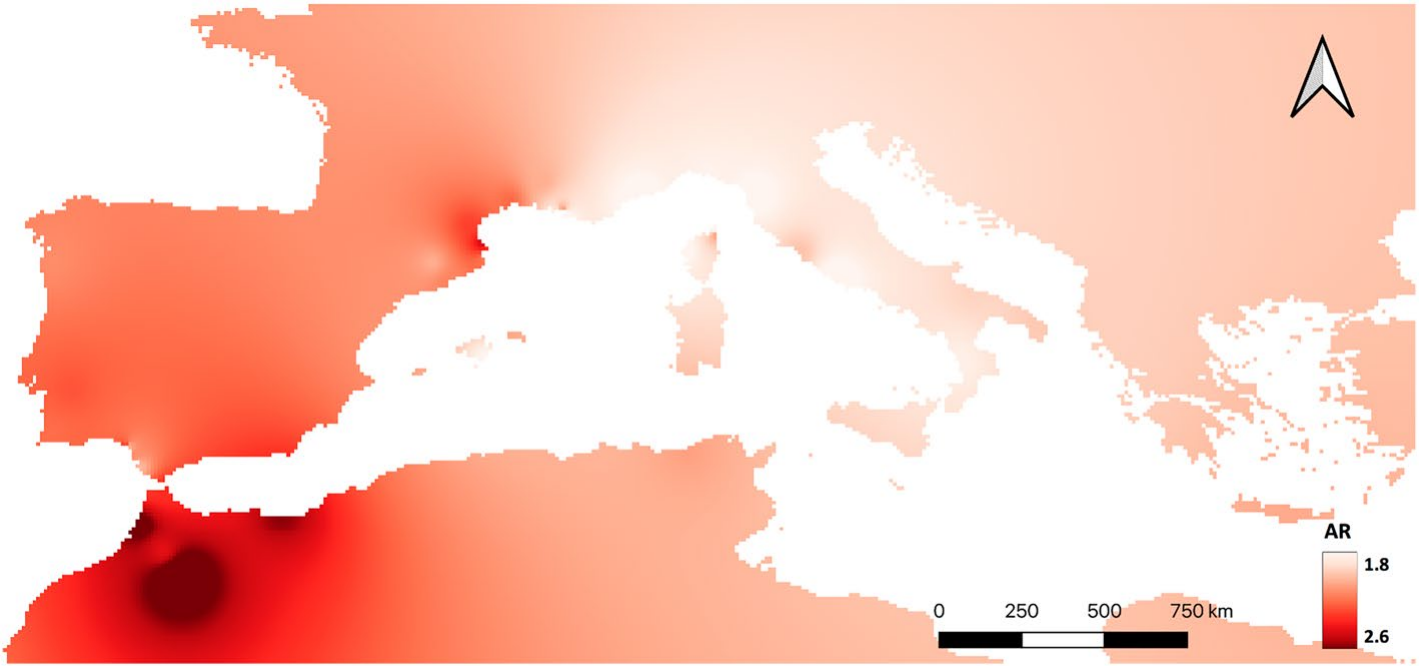
Inverse distance weighted (IDW) interpolation of the rarefied Allelic richness (R-Ar) for the 44 populations of *T. mauritanica* used in this study. The spatial interpolation is displayed in a red (high) to white (low) gradient. Map generated using QGIS software^95^.

Regarding the genetic diversity results, Morocco (the highest) and the Iberian Peninsula are clearly the regions harbouring the populations containing the uppermost number of alleles and allelic richness, in contrast to Greece and the Balearic Islands (Table 1 and S3). Indeed, the spatial interpolation of the rarefied allelic richness, identifies Morocco, the Iberian Peninsula and also the west-Mediterranean coast of France as major centres of genetic diversity for *T. mauritanica* (Fig. 2).

**Table 1:** Genetic diversity statistics calculated from the microsatellite markers for each major geographic region (see “Methods” section).

Population	N	N_a_	Ar	R-Ar	H_o_	H_e_
Corsica	20	22	2.25	**1.97**	0.31	0.40
France	55	39	2.70	2.17	0.35	0.46
Greece	**6**	**20**	**2.20**	2.03	**0.28**	**0.39**
Morocco	46	**85**	**4.85**	**3.37**	**0.62**	**0.77**
Iberia	**224**	56	3.07	2.37	0.46	0.54
Balearic Islands	18	28	2.49	2.07	**0.28**	0.41
Tunisia	17	27	2.67	2.20	0.38	0.47
Madeira	60	31	2.71	2.17	0.36	0.48
Italy	108	29	2.46	2.08	0.35	0.46

Minimum and maximum values are highlighted in bold.

The following parameters are displayed: sample size (N), mean number of alleles (N_a_), allelic richness (Ar), rarefied allelic richness (R-Ar), observed heterozygosity (H_o_) and expected heterozygosity (H_e_).

**Figure 3:**
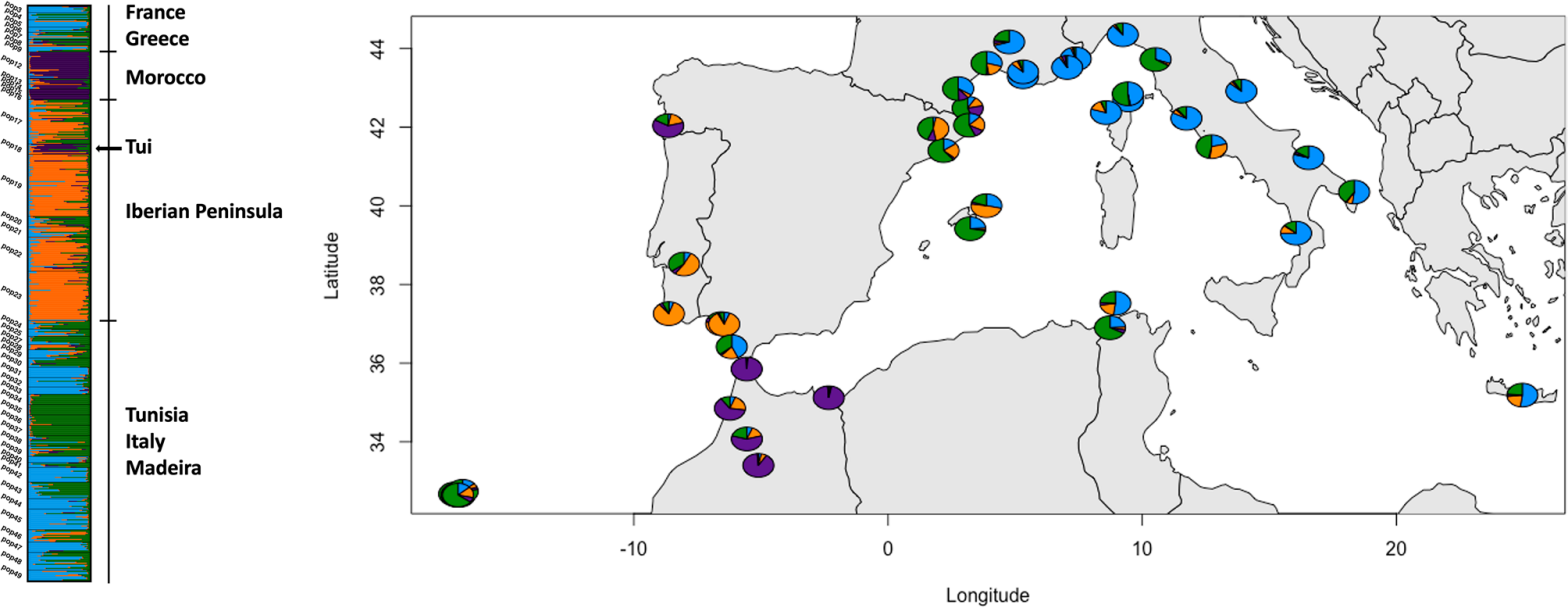
On the left is represented STRUCTURE’s bar plot displaying the assignment of individuals for the best K (K = 4; but see Fig. S1). Individuals are grouped by populations displayed on the left of the bar plot. Population codes are denoted in Table S1 and STRUCTURE analysis within each major geographic group is displayed in Figs. S2–S4. On the right figure, the pie charts display population averages of ancestry proportions according to STRUCTURE results. Geographic plotting was performed using the R package *mapplots*^102^.

**Figure 4:**
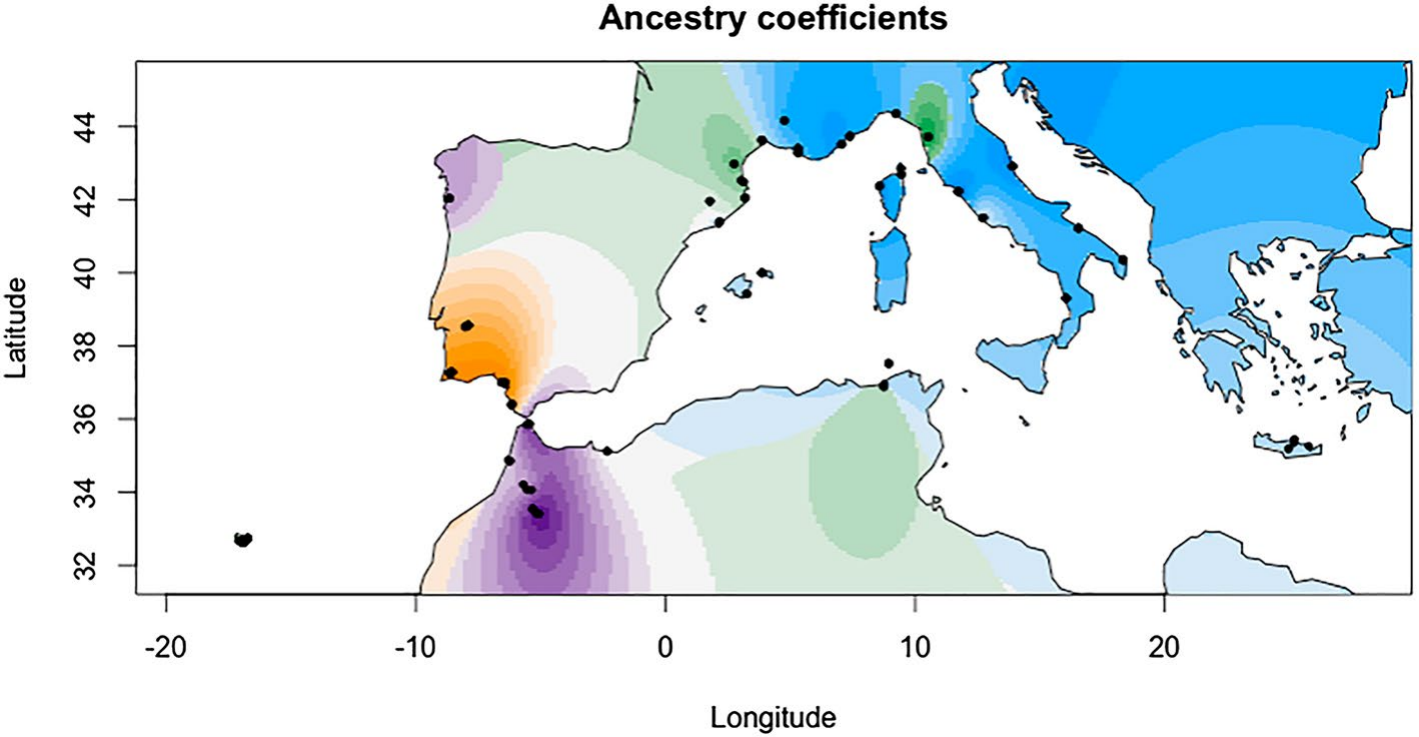
Geographic maps of ancestry coefficients using K = 4 ancestral populations (see STRUCTURE results), obtained with tess3r. Colours match with STRUCTURE genetic clusters. Geographic plotting of ancestry coefficients was performed using the R package *mapplots*^102^.

**Figure 5:**
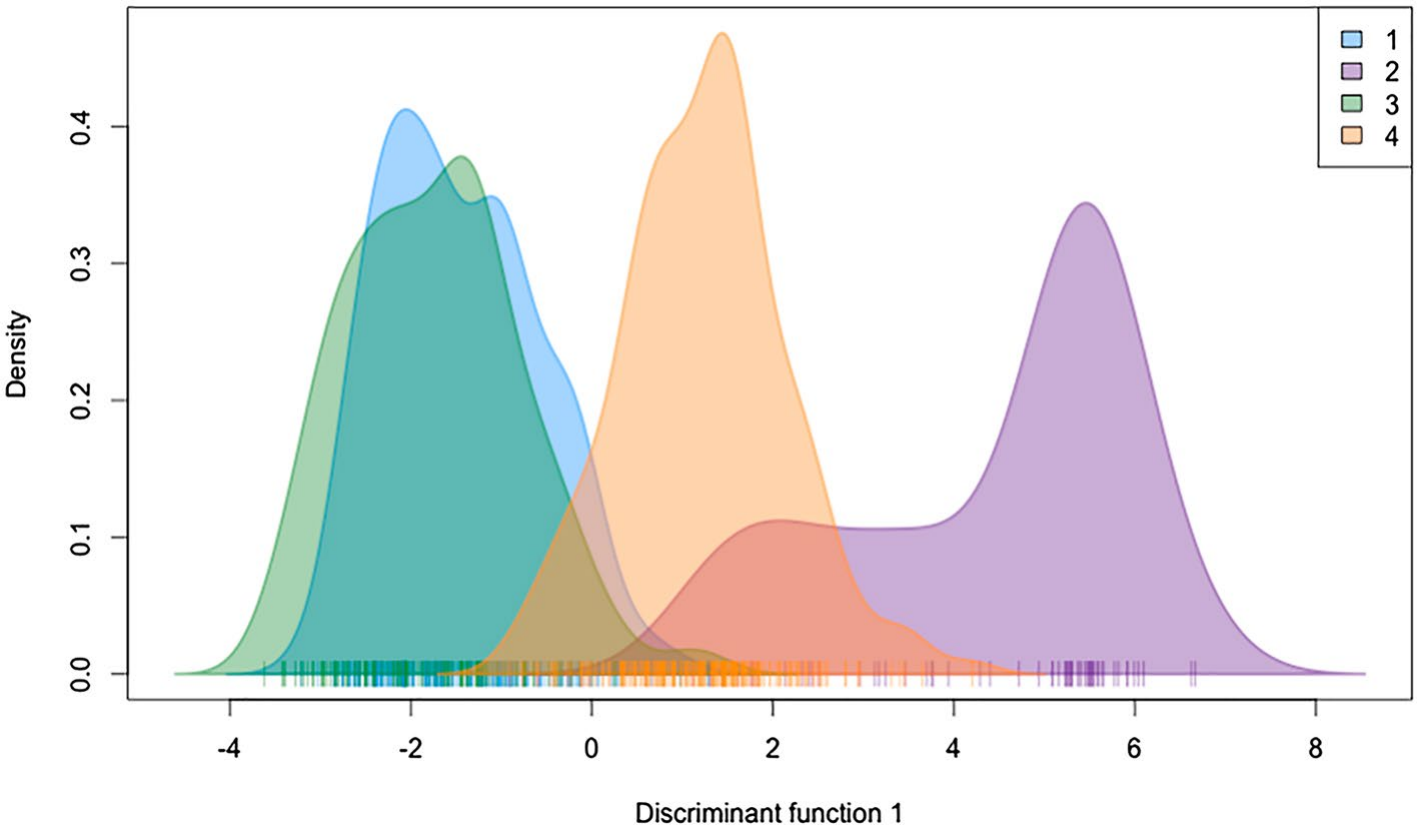
Relative densities of genotyped individuals plotted against the discriminant function 1 from the Discriminant analysis of principal components (DAPC), representing genetic divergence among groups. Colours match with STRUCTURE genetic clusters.

According to Evanno et al.^45^’s method, STRUCTURE identified that the best K was K = 4 (Fig. 3 and Fig. S1), but also both *tess3r* and DAPC seem to indicate that the genetic diversity of *T. mauritanica* could be substructured into four clusters (Figs. S2 and S3), since there is a slight elbow/plateau in both cross-validation and BIC values when the number of clusters is 4. Nevertheless, one should always be careful about over-interpreting these graphs and the value of K, since the number of genetic groups detected by ancestry estimation programs does not necessarily correspond to the number of biologically meaningful populations in the sample^46^. Hence, the major identified geographic groups are France and Greece, Morocco, the Iberian Peninsula, and Tunisia, Italy and Madeira. This geographic sub-structuring is also evident with tess3r (Fig. 4). Morocco and the Iberian Peninsula present unique genetic patterns, with very little allele sharing with other geographic regions (Figs. 3, 4, 5 and S4). The only exception concerns the individuals from the Tui population (Spain) that share most of their alleles with Morocco, instead of the remaining Iberian populations. Additionally, the DAPC evidence that the genetic clusters comprising the Iberian Peninsula and Morocco populations are genetically unique and divergent from the other groups (Figs. 5 and S4). On the contrary, clusters 1 and 3 are overlapped. Moreover, the results from both F_st_ and G’’_st_ support most of all defined geographic regions as independent units (Tables S4 and S5). The only exceptions were the Greece-Corsica and Corsica-Italy combinations.

**Figure 6:**
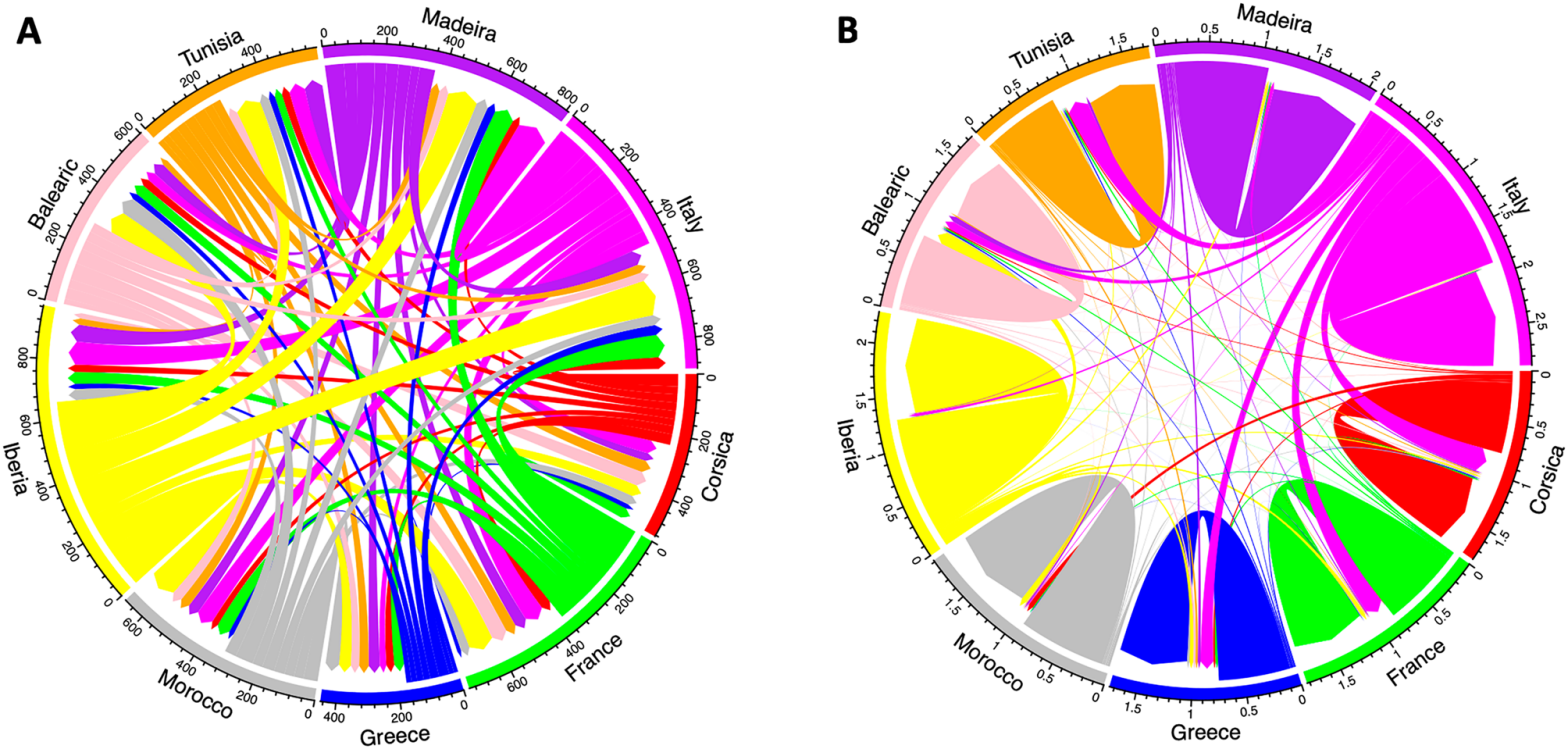
Gene flow diagrams for the Moorish gecko among different geographic regions, corresponding to (**A**) historical gene flow estimates from Migrate-n, and (**B**) contemporary gene flow estimates from BayesAss. Grid width represents the total amount of incoming and outgoing gene flow estimated for each population. Arrows indicate the direction of gene flow among the populations while the width of arrows is proportional to the relative amount of gene flow observed among connected populations. Exact gene flow estimates are presented in Supplementary Tables S6 and S7, respectively.

Finally, the results from the gene flow assessment suggest that the direction and intensity of colonization by the Moorish gecko, have changed over time (Fig. 6). Currently, most of the gene flow occurs within each geographic range, and Italy acts as the main source of introduced individuals into new areas. Quite the reverse, in the past there was little intra-population migration and massive inter-population gene flow, with the Iberian Peninsula and at some point, also Italy being the main geographic sources of introduction. However, both these regions were simultaneously hosts of introduced individuals from different origins, and in most geographic territories the incoming and outcoming gene flow were 50/50.

From the comparison of both gene flow diagrams, it is also evident that regions such as Greece, France, Corsica, the Balearic Islands and Tunisia, have been the stage of multiple introductions from Italy in two different moments in time.

## Discussion

Accumulated genetic evidence suggests that species introductions are often featured by complex histories, but also underlines that several key and relatively simple components may be part of the process^47^.

The Mediterranean region is a world biodiversity hotspot^48^ with one of the longest histories of interaction between humans and biodiversity, with multiple introductions of taxa occurring over millennia^49^. In that sense, humans have been key dispersal drivers of several alien reptiles in this region. However, their distributions are determined by a complex interplay between human activities, geographic factors and species traits^50^.

A common finding in the Mediterranean region is that many introduced populations may originate from multiple geographically distinct sources e.g.,^8,51,52^. Alternately, and a common scenario for the origin of multiple populations is a serial founding from a single source e.g.,^8,53,54^. Another scenario which is rarely considered is a model concerning multiple introductions from the same source in different points in time.

The Balearic Islands appear as a striking example of a region marked by several faunal introductions across time; this archipelago that once harboured substantial levels of endemicity, now hosts more alien than native reptile and amphibian taxa reviewed in^55,56^. The same occurs nowadays in Madeira Island, where there is a single endemic lizard species (*Teira dugesii*), surpassed by two introduced geckos, *Hemidactylus mabouia*, and *Tarentola mauritanica*^10,34,57^, one snake (Ramphotyphlops braminus;^58^), and one allochthonous skink (Chioninia fogoensis;^59^). Hence, it is clear that although largely sedentary, reptiles are frequently introduced by humans, many times during transport of building material, soil or cultivated plants^4^.

*Tarentola* geckos in particular, which are primarily a North African clade, have naturally reached long distances such as many Macaronesia islands but also Cuba and the Bahamas, most likely by rafting on buoyant vegetation, at least 23 Mya^42^. Nevertheless, the current geographic distribution of *T. mauritanica* is partly the result of recurrent anthropogenic introductions e.g.,^22–26,28–30,34,36,37,39,41,42,60^. Their success as human-assisted colonizers is somewhat associated with the synanthropic habits of this species^61^, allied with their relatively small size and cryptic nature.

Our results suggest that Morocco represents the genetic diversity hotspot for the European lineage of the Moorish gecko, followed by the Iberian Peninsula (Table 1 and Fig. 2). This is indeed not a surprise considering the Moroccan origin of *T. mauritanica*, harbouring most of the mitochondrial DNA diversity as well^23–25,43^. Although, the mtDNA results from Belluardo et al.^44^ suggest Italy as the centre of diversification of the European lineage of *T. mauritanica*, we have to acknowledge that their North African sampling was very limited and, possibly unable to tackle its underlaying genetic diversity. Moreover, all population structure analyses presented here support Morocco and the Iberian Peninsula as two divergent clusters with very little gene flow between them or with the other remaining groups (Figs. 3, 4, 5). The only exception is the Tui population, whose individuals share most of their alleles with the Moroccan populations, evidencing a clear case of significant introduction into this Spanish locality (Fig. 3). The two other groups comprising France, Italy, Tunisia, Madeira and Greece are undoubtedly admixed. The historical literature in France seems to indicate a maritime transport from North Africa as the source of introduction of *T. mauritanica*, based on its presence in material freshly landed from Algeria in the port of Sète^62^. However, an earlier account by Crespon^63^, reports that the species is rarer in south-west France than in the Provence region, which borders Italy. His earlier accounts, therefore, also point to a historically important arrival from Italy, which is supported by the gene flow results obtained here (Fig. 6). Although the current study does not include specimens from Algeria, the results from Fig. 6A indicate the existence of historical gene flow from both Morocco and Tunisia into France. Hence, it is plausible to assume that if Algeria had been sampled, we might also detect introductions from here to France, as has already been described for two amphibian species, *Discoglossus pictus*^64^ and *Pelophylax saharicus*^65^.

Considering the distribution of the genetic diversity but mostly of the evolutionary history of this European lineage, a bigger contribution of Morocco as a source of introduction was expected (Fig. 6). Indeed, the historical gene flow estimates identify the Iberian Peninsula as the major contributor of colonizing individuals, and also Italy in a smaller scale. According to Rato et al.^25^, the diversification of the European lineage started around 2.47Mya, most likely in Morocco. This means that the colonization of the Northern Mediterranean from North Africa could be quite ancient, and if so, maybe impossible to be detected using fast-evolving markers, such as microsatellites. In fact, the genus *Tarentola* and *T. mauritanica*, or morphologically closely-related taxa, were identified in the fossil records from Spain dated from the Early Pleistocene^66^, supporting the ancient occurrence of this taxon in the Iberian Peninsula. As the high incidence of microsatellite homoplasy increases with evolutionary distance, it might limit the depth of the phylogeny at which it is possible to make inferences^67^. Therefore, the obtained ancient gene flow scenario matches better with the known human history in the Mediterranean during the Classical Era (600 B.C. to A.D. 476), which highlights the importance of the Iberian Peninsula and Italy. This was the time of the Roman Empire (753 B.C. to A.D. 476) when cities were being developed, and communication networks expanded, which have clearly favoured the translocation of highly anthropophilic species^68^. In fact, during this historical time there was a peak of introduced reptile and amphibian species in the Iberian Peninsula^68^, a region known to be one of the most important gold suppliers during the Roman Empire^69^. Hence, these results together with the distribution of the genetic diversity support the Iberian Peninsula as the most likely entering point of the European lineage of *T. mauritanica* from North Africa, and from there to the remaining geographic regions.

Very importantly, we need to acknowledge Sardinia and Sicily as major sampling gaps in this study. Apart from being the two biggest islands in the Mediterranean, they had many historical connections with North Africa including herpetological species exchange^70,71^. The study from Belluardo et al.^44^ identified the presence of a common widespread mitochondrial 16S haplotype in Sardinia, Sicily, Morocco, Algeria and Tunisia. Additionally, fossil remains of *T. mauritanica* dated from the Late Pleistocene-Holocene were found in Sicily (San Vito lo Capo)^66^. However, the origin of both Sardinian and Sicilian populations remains unknown, and an excellent opportunity for future studies.

Currently, Italy seems to be the main source of Moorish gecko introduced individuals, although most of the gene flow takes place within each geographic region, contrary to the long-distance colonization typical in *Tarentola* geckos^42^. Accumulating evidence is strongly identifying the “plant nursery trade” (commerce in live plants for ornamental purposes) as one of the main forms of reptile introduction into new territories^4^, since these animals often use plants and trees for refuge and thermoregulation e.g.,^72,73^. This kind of human-mediated trade in the Mediterranean has been responsible, for instance, for the introduction of the Italian wall lizard, *Podarcis siculus* e.g.,^8,74^, the brahminy blind snake, *Indotyphlops braminus*^75^, and the colubrid snakes *Hemorrhois hippocrepis*, *Malpolon monspessulanus*, and *Zamenis scalaris*^53^, outside their native geographic ranges. Curiously, many of these introductions result from the trade of old olive trees transported from Italy^76–78^. Indeed, the olive tree trade appears as a modern vector for bioinvasions across the Mediterranean for a wide spectrum of alien species, *Tarentola mauritanica* included, having been recorded in Catalonia (Spain) around olive trees brought from Italy^77^, but also in Lake Garda, Northern Italy^79^, and Beaugeay, on the Atlantic coast of France^80^. Hence, previous studies together with the results obtained here, point to the plant nursery trade from Italy as a putative modern route responsible for the introduction of *T. mauritanica* across a wide geographic range. Additionally, the Mediterranean Basin has a very intense maritime traffic (especially since the opening of the Suez Canal connecting the Mediterranean and the Red Sea^81^), which is thought to be the main driver for the introduction of Italian Moorish gecko individuals in the islands of Corfu^30^ and Lesvos^31^ in Greece. Overall, the colonization of the European lineage of *T. mauritanica* in the Mediterranean and Macaronesia seems to result from a combination of several human-mediated introductions from multiple geographic sources, with many regions having been colonized by Italy in at least two different periods far apart in time. Most importantly, this study has identified and highlighted the most likely current colonization routes for this lineage, which will hopefully help authorities in the design of better monitoring and inspection conservation programs.

## Materials and methods

### Study sites and sampling

In this study, 555 individuals of *Tarentola mauritanica* collected across 44 Mediterranean (some islands included) and Madeira populations were used, with each location represented by at least five individuals. Genotypes from all Italian populations were retrieved from Belluardo et al.^44^. All individuals included in this study belong to the mitochondrial European clade^22–26,44,82,83^. Tissue from tail tip muscle was collected from each individual and preserved in 96% ethanol. More details on the localities and origin of the samples are presented in Table S1 and Fig. 1.

### DNA extraction and microsatellite genotyping

Genomic DNA was extracted using a standard high-salt protocol^84^. 11 microsatellite loci were genotyped (9 dinucleotide, and 2 trinucleotide repeats) from a battery developed specifically for *T. mauritanica* (Mt3, Mt6, Mt7, Mt11, Mt13, Mt14, Mt16, Mt21, Mt24, Mt27, and Mt29)^85^. All loci were amplified according to the described conditions in Arranz et al.^85^. All amplifications were performed including negative controls. PCR products were separated by size on an ABI3130xl genetic analyser using the 350ROX size standard. Allele sizes were determined using GENEMAPPER v.6.0^86^ and checked manually.

### Data screening and quality assessment

The presence of possible null alleles, allele scoring errors due to stuttering and large allele dropout was evaluated using MICRO-CHECKER v2.2.3^87^. Linkage disequilibrium (LD) and deviations from Hardy–Weinberg equilibrium (HWE) were tested with “GENEPOP on the Web” (dememorization = 1000; batch number = 100; iteractions per batch = 1000)^88,89^. We applied the False Discovery rate^90^ to correct *p* values (*p* < 0.05) from HWE and LD multiple exact tests, using the package *fdrtool*^91^ in R^92^.

To avoid the computer intensive assessment of such a large number of distinct populations, these were grouped into nine geographical regions (Iberian Peninsula, Corsica, Continental France, Greece, Morocco, Balearic Islands, Tunisia, Madeira and continental Italy), which were considered in some of the posterior analyses (see population structure results that support this).

### Genetic variation

Basic microsatellite diversity was evaluated separately for each population based on the number of alleles per locus (Na), expected and observed heterozygosity (H_exp_ and H_obs_, respectively), using the R *diveRsity* package and the *divBasic* function^93^. The rarefied allelic richness (R-Ar) per population was calculated with the R package *hierfstat*^94^, which accounts for variation in sample size (each sample included a minimum of five loci). To identify geographic changes in genetic diversity, the rarefied allelic richness was spatially interpolated using the Inverse Distance Weighting method (IDW) in QGIS v.3.16.11 “Hannover”^95^.

### Population structure

Population differentiation was assessed by means of pairwise F_ST_^96^ and G”_ST_^97^ measures, using the *diffCalc* function from the R *diveRsity* package. Respective 95% confidence intervals (CIs) were estimated using 1000 permutations, and pairwise estimates were considered significant when 95% CIs did not overlap zero. These analyses were applied to all 44 populations and also to the nine defined geographic regions.

Genetic structure was assessed using a Bayesian clustering analysis, implemented in STRUCTURE 2.3.4^98,99^. Ten independent runs were performed for a number of clusters (K) ranging between 2 and 8. Runs consisted of a burn-in period of 10^4^ iterations, followed by 10^6^ MCMC reps, correlated allele frequencies, admixture model, and no prior information regarding population of origin. The best K was identified using STRUCTURE HARVESTER Web v0.6.94^45,100^, and deemed as the best K describing the observed genetic data. Graphical plotting of STRUCTURE results was implemented in the online software CLUMPAK^101^, and geographic plotting of ancestry coefficients using the R package *mapplots*^102^.

Additionally, two other methods were implemented as alternative and complementary approaches to assess the genetic diversity; a Bayesian Clustering algorithm using tessellations and Markov models for spatial population genetics under the R package *tess3r*^103^; and a discriminant analysis of principal components DAPC;^104^.

The *tess3r* algorithm implements a new version of the program TESS^105^, based on geographically constrained matrix factorization and quadratic programming techniques. The new algorithms are several orders faster than the Monte-Carlo algorithms implemented in previous versions of TESS.

The DAPC summarizes the data to minimize genetic differentiation within previously defined groups, while maximizing it between groups, and it does not consider HWE or linkage equilibrium as necessary conditions. For this analysis, we assumed four genetic clusters based on the Bayesian Information Criterion (BIC) and ΔK (see “Results” section), while the number of retained principal components and discriminant functions were selected following the package’s manual guidelines to avoid overfitting.

### Gene flow assessment

Historical and contemporary migration rates were estimated among the major nine geographic regions with MIGRATE v3.7.2^106^ and BayesAss v3.04 (BA3) (Wilson and Rannala, 2003), respectively.

MIGRATE uses the coalescent in a Bayesian or maximum likelihood framework to calculate two parameters from the data, θ and M, where θ represents the effective population size (4Neμ for nuclear DNA), and M the mutation-scaled immigration rate (m/µ). This coalescent-based approach is most suitable for estimating migration rates over thousands of years or approximately 4N_e_ generations in the past^107^. The data were assumed to follow a Brownian motion mutation model. The F_ST_ calculation method was used to generate starting values for both θ and M. Uniform priors were specified for both parameters with a minimum of 0, mean of 50, maximum of 100, and a delta of 10. The Bayesian method was implemented to infer θ and M, specifying two independent runs, static heating with four chains (temperatures: 1.0, 1.5, 3.0, 10,000.0), a sampling increment of 20, 50,000 recorded steps, and a burn-in of 10,000. Convergence was assessed by examination of ESS values with a target of at least 1000.

Unlike MIGRATE, BAYESASS does not assume genetic equilibrium and is therefore, more suitable for inferring contemporary (over the past few generations) processes. The model in BAYESASS assumes linkage equilibrium between loci but allows for deviations in Hardy–Weinberg proportions by introducing an additional inbreeding (F) parameter. Several analyses with different starting seeds were performed and each Markov chain Monte Carlo run involved 10^8^ iterations and discarding of the first 10^6^ iterations as burn-in. The delta values DA, DF, and DM were set to 0.4, 0.5, and 0.4, respectively. Convergence of the chains was validated using Tracer v1.7.1^108^.

Graphical plotting of migration rates among geographical regions was performed with the R package *circlize*^109^.

### Ethics declaration

All methods were carried out in accordance with relevant guidelines and regulations. The entire experimental protocol was approved by the Ethics Committee of the University of Porto (https://www.up.pt/portal/pt/conhecer/organizacao/comissao-de-etica/). Codes of all issued sampling permits are provided in Table S1. No humans were part of this study. All work was conducted in accordance with ARRIVE guidelines.

### Supplementary Information

Supplementary Information.

## Data Availability

Individual genotypes generated during the current study are available in Figshare repository (https://doi.org/10.6084/m9.figshare.24105732.v1).

## References

[1] David P (2017). Impacts of invasive species on food webs: A review of empirical data. Adv. Ecol. Res..

[2] Keller RP, Geist J, Jeschke JM, Kühn I (2011). Invasive species in Europe: Ecology, status, and policy. Environ. Sci. Eur..

[3] Simberloff D (2013). Impacts of biological invasions: What’s what and the way forward. Trends Ecol. Evol..

[4] Kraus F (2009). Alien Reptiles and Amphibians: A Scientific Compendium and Analysis.

[5] Dlugosch KM, Parker IM (2008). Founding events in species invasions: Genetic variation, adaptive evolution, and the role of multiple introductions. Mol. Ecol..

[6] Lodge DM (2006). Biological invasions: Recommendations for US policy and management. Ecol. Appl..

[7] Estoup A, Guillemaud T (2010). Reconstructing routes of invasion using genetic data: Why, how and so what?. Mol. Ecol..

[8] Silva-Rocha I, Salvi D, Carretero M (2012). Genetic data reveal a multiple origin for the populations of the Italian wall lizard *Podarcis sicula* (Squamata: Lacertidae) introduced in the Iberian Peninsula and Balearic islands. Ital. J. Zool..

[9] Salvi D, Harris DJ, Perera A, Bologna MA, Carretero MA (2011). Preliminary survey on genetic variation within the Pygmy Algyroides, *Algyroides fitzingeri*, across Corsica and Sardinia. Amphibia-Reptilia.

[10] Rato C, Martins B, Rocha R, Silva-Rocha I (2021). Uncovered genetic diversity in *Hemidactylus mabouia* (Reptilia: Gekkonidae) from Madeira Island reveals uncertain sources of introduction. Amphibia-Reptilia.

[11] Arthofer W, Heussler C, Krapf P, Schlick-Steiner BC, Steiner FM (2018). Identifying the minimum number of microsatellite loci needed to assess population genetic structure: A case study in fly culturing. Fly.

[12] Villanueva B, Verspoor E, Visscher P (2002). Parental assignment in fish using microsatellite genetic markers with finite numbers of parents and offspring. Anim. Genet..

[13] Guichoux E (2011). Current trends in microsatellite genotyping. Mol. Ecol. Resour..

[14] Wenburg JK, Bentzen P, Foote CJ (1998). Microsatellite analysis of genetic population structure in an endangered salmonid: The coastal cutthroat trout (*Oncorhynchus clarki clarki*). Mol. Ecol..

[15] Guo X-Z (2016). Phylogeography and population genetics of *Schizothorax o’connori*: Strong subdivision in the Yarlung Tsangpo River inferred from mtDNA and microsatellite markers. Sci. Rep..

[16] Kleinhans C, Willows-Munro S (2019). Low genetic diversity and shallow population structure in the endangered vulture, *Gyps coprotheres*. Sci. Rep..

[17] Bonato L (2018). Diversity among peripheral populations: Genetic and evolutionary differentiation of *Salamandra atra* at the southern edge of the Alps. J. Zool. Syst. Evol. Res..

[18] Frantz A, Cellina S, Krier A, Schley L, Burke T (2009). Using spatial Bayesian methods to determine the genetic structure of a continuously distributed population: Clusters or isolation by distance?. J. Appl. Ecol..

[19] Balkenhol N (2014). A multi-method approach for analyzing hierarchical genetic structures: A case study with cougars *Puma concolor*. Ecography.

[20] Kobayashi T, Sota T (2019). Contrasting effects of habitat discontinuity on three closely related fungivorous beetle species with diverging host-use patterns and dispersal ability. Ecol. Evol..

[21] Vogrin, M. *et al. Tarentola mauritanica*. The IUCN Red List of Threatened Species 2017: e.T61578A63716927. 10.2305/IUCN.UK.2017-2.RLTS.T61578A63716927.en. Downloaded on 23 February 2021 (2017).

[22] Perera A, Harris DJ (2008). Genetic diversity in the gecko *Tarentola mauritanica* within the Iberian Peninsula. Amphibia-Reptilia.

[23] Harris DJ, Batista V, Lymberakis P, Carretero MA (2004). Complex estimates of evolutionary relationships in *Tarentola mauritanica* (Reptilia: Gekkonidae) derived from mitochondrial DNA sequences. Mol. Phylogenetics Evol..

[24] Harris DJ, Batista V, Carretero MA, Ferrand N (2004). Genetic variation in *Tarentola mauritanica* (Reptilia: Gekkonidae) across the Strait of Gibraltar derived from mitochondrial and nuclear DNA sequences. Amphibia-Reptilia.

[25] Rato C, Carranza S, Harris DJ (2012). Evolutionary history of the genus *Tarentola* (Gekkota: Phyllodactylidae) from the Mediterranean Basin, estimated using multilocus sequence data. BMC Evol. Biol..

[26] Rato C, Carranza S, Perera A, Carretero MA, Harris DJ (2010). Conflicting patterns of nucleotide diversity between mtDNA and nDNA in the Moorish gecko, Tarentola mauritanica. Mol. Phylogenetics Evol..

[27] Bailon, S. & Rage, J. Données fossiles et mise en place de l’herpétofaune actuelle de la France. *Atlas des Amphibiens et Reptiles de France. Biotope & Muséum national d’Histoire naturelle, Paris. Coll. Inventaires & biodiversité* 33–39 (2012).

[28] Deso G (2020). Documenting the introduction of the Moorish gecko *Tarentola mauritanica* (Linnaeus, 1758) (Squamata: Phyllodactylidae) on the Levant and Port-Cros Islands (Hyères Archipelago, Var department, France). Herpetol. Notes.

[29] Rato C (2021). Alborán Island, a small meeting point for three invasive lizards, whose geographic origin is uncovered by molecular analysis. BioInvasions Rec..

[30] Mačát Z, Starcová M, Červenka J, Jablonski D, Šandera M (2014). A molecular assessment and first record of *Tarentola mauritanica* (Squamata: Phyllodactylidae) on Corfu, Greece. Salamandra.

[31] Mizerakis V, Strachinis I (2017). New record of *Tarentola mauritanica* (Squamata: Phyllodactylidae) from Lesvos island, Greece. Herpetol. Notes.

[32] Strachinis I, Artavanis D (2017). Additions to the known herpetofauna of the Island of Ithaki, Ionian Sea, Greece. Herpetozoa.

[33] Strachinis I, Lymberakis P, Tzoras E (2023). *Tarentola mauritanica* (Squamata: Phyllodactylidae) in Greece: An update on the species’ distribution, including new records. Ecol. Balk..

[34] Báez, M. & Biscoito, M. in *First symposium of fauna and flora of the Atlantic islands.*

[35] Rato C, Resendes R, Tristão da Cunha R, Harris DJ (2015). First record of *Tarentola substituta* Joger, 1984, and genetic identification of *Tarentola mauritanica* (Linnaeus, 1758) in the Azores. Herpetozoa.

[36] Ortiz-Medina JA, Cabrera-Cen DI, Chan-Noh MM, Cedeño-Vázquez JR (2019). First record of the Moorish Gecko, *Tarentola mauritanica* (Linnaeus, 1758) (Squamata: Phyllodactylidae), Mexico. Herpetol. Notes.

[37] Díaz-Fernández L, Paz A, Valdecantos S (2019). First checked arrival of *Tarentola mauritanica* (Linnaeus, 1758) in Salta, Argentina (Squamata; Phyllodactylidae). Herpetol. Notes.

[38] Mahrdt CR (1998). Geographic distribution of *Tarentola mauritanica*. Herpetol. Rev..

[39] Arredondo C, Núñez H (2014). *Tarentola mauritanica* (Linnaeus, 1758), a new species of lizard for Chile (Reptilia, Phyllodactylidae). Boletín del Museo Nacional de Historia Natural.

[40] Baldo D, Borteiro C, Brusquetti F, García JE, Prigioni C (2008). Reptilia, Gekkonidae, *Hemidactylus mabouia*, *Tarentola mauritanica*: Distribution extension and anthropogenic dispersal. Check List.

[41] Huerta-Vera S (2016). Registros de Gecko Mediterráneo, *Tarentola mauritanica* (Linnaeus 1758) (Squamata, Phyllodactylidae), en zona semi-urbana de Peñalolén, Región Metropolitana. Boletín Chileno de Herpetología.

[42] Carranza S, Arnold EN, Mateo JA, López-Jurado LF (2000). Long-distance colonization and radiation in gekkonid lizards, *Tarentola* (Reptilia: Gekkonidae), revealed by mitochondrial DNA sequences. Proc. R. Soc. Lond. B.

[43] Rato C, Harris DJ, Carranza S, Machado L, Perera A (2016). The taxonomy of the *Tarentola mauritanica* species complex (Gekkota: Phyllodactylidae): Bayesian species delimitation supports six candidate species. Mol. Phylogenetics Evol..

[44] Belluardo, F. *et al.* Multilocus genetic assessment of the *Tarentola mauritanica* clade III in the Italian Peninsula and main islands: new insights into the evolutionary history of the clade. *J. Zool. Syst. Evol. Res. *submitted (2023).

[45] Evanno G, Regnaut S, Goudet J (2005). Detecting the number of clusters of individuals using the software STRUCTURE: A simulation study. Mol. Ecol..

[46] François O, Durand E (2010). Spatially explicit Bayesian clustering models in population genetics. Mol. Ecol. Resour..

[47] Guillemaud T, Ciosi M, Lombaert E, Estoup A (2011). Biological invasions in agricultural settings: Insights from evolutionary biology and population genetics. C. R. Biol..

[48] Myers N, Mittermeier RA, Mittermeier CG, da Fonseca GAB, Kent J (2000). Biodiversity hotspots for conservation priorities. Nature.

[49] Blondel J, Aronson J, Bodiou J-Y, Boeuf G (2010). The Mediterranean Region: Biological Diversity in Space and Time.

[50] Silva-Rocha IR, Salvi D, Carretero MA, Ficetola GF (2019). Alien reptiles on Mediterranean Islands: A model for invasion biogeography. Divers. Distrib..

[51] Santos JL (2019). Phylogeographic evidence for multiple long-distance introductions of the common wall lizard associated with human trade and transport. Amphibia-Reptilia.

[52] Graciá E (2017). Human-mediated secondary contact of two tortoise lineages results in sex-biased introgression. Sci. Rep..

[53] Silva-Rocha I, Salvi D, Sillero N, Mateo JA, Carretero MA (2015). Snakes on the Balearic Islands: An invasion tale with implications for native biodiversity conservation. PLoS ONE.

[54] Graciá E (2017). Expansion after expansion: Dissecting the phylogeography of the widely distributed spur-thighed tortoise, *Testudo graeca* (Testudines: Testudinidae). Biol. J. Linn. Soc..

[55] Pinya S, Carretero MA (2011). The Balearic herpetofauna: A species update and a review on the evidence. Acta Herpetol..

[56] Silva-Rocha I (2018). Herpetological history of the Balearic Islands: When aliens conquered these islands and what to do next. Hist. Bioinvasions Mediterr..

[57] Jesus J, Freitas AI, Brehm A, Harris J (2002). An introduced population of *Hemidactylus mabouia* (Moreau de Jonnés, 1818) on Madeira Island. Herpetozoa.

[58] Jesus J, Goncalves R, Spinola C, Brehm A (2013). First record of *Ramphotyphlops braminus* (Daudin, 1803) on Madeira Island (Portugal). Herpetozoa.

[59] Clemens DJ, Allain SJ (2020). First evidence of Fogo Island skink (*Chioninia fogoensis*) introduction to the island of Madeira. Herpetol. Bull..

[60] Delaugerre M-J, Thibault J-C, Beuneux GL (2017). Renouvellement récent des faunes de vertébrés sur l’île de Cavallo (archipel des Lavezzi, Corse). Ecol. Mediterr..

[61] Arnold EN, Ovenden DW (2002). A Field Guide to the Reptiles and Amphibians of Britain and Europe.

[62] Mayet V (1898). in *Géographie générale du dépatement de l’hérault*. Tome.

[63] Crespon, J. *Faune méridionale ou description de tous les animaux vertébrés vivants et fossiles, sauvages ou domestiques qui se rencontrent toute l’année ou qui ne sont pas de passage dans la plus grande partie du Midi de la France; suivie d’une méthode de taxidermie ou l’art d’empailler les oiseaux. Tome deuxiéme* (1844).

[64] Lanza B, Nascetti G, Capula M, Bullini L (1986). Les Discoglosses de la région méditerranéenne occidentale (Amphibia; Anura; Discoglossidae). Bull. de la Société Herpétologique de France.

[65] Doniol-Valcroze P (2021). Discovery of a *Pelophylax saharicus* (Anura, Ranidae) population in Southern France: A new potentially invasive species of water frogs in Europe. Amphibia-Reptilia.

[66] Villa A, Delfino M (2019). Fossil lizards and worm lizards (Reptilia, Squamata) from the Neogene and Quaternary of Europe: An overview. Swiss J. Palaeontol..

[67] Jarne P, Lagoda PJ (1996). Microsatellites, from molecules to populations and back. Trends Ecol. Evol..

[68] Santos X (2015). Síntesis de las introducciones de anfibios y reptiles en España. Boletín de la Asociación Herpetológica Española.

[69] Martins CMB (2008). A exploração mineira romana e a metalurgia do ouro em Portugal.

[70] Stöck M (2016). On the origin of the recent herpetofauna of Sicily: Comparative phylogeography using homologous mitochondrial and nuclear genes. Zoologischer Anzeiger-J. Comp. Zool..

[71] Pous, P. D., Speybroeck, J., Bogaerts, S., Pasmans, F. & Beukema, W. A contribution to the atlas of the terrestrial herpetofauna of Sardinia (2012).

[72] Vroonen J, Vervust B, Fulgione D, Maselli V, Van Damme R (2012). Physiological colour change in the Moorish gecko, *Tarentola mauritanica* (Squamata: Gekkonidae): Effects of background, light, and temperature. Biol. J. Linn. Soc..

[73] Mezzasalma, M. *et al.* in *Atti VIII Congresso Nazionale Societas Herpetologica Italica* (eds L. Di Tizio, A.R. Di Cerbo, N. Di Francesco, & A. Cameli) 129–133 (lanieri Edizioni, Pescara, Chieti, 2010).

[74] Silva-Rocha I (2014). Molecular assessment of *Podarcis sicula* populations in Britain, Greece and Turkey reinforces a multiple-origin invasion pattern in this species. Acta Herpetol..

[75] Rato C (2015). A molecular assessment of European populations of *Indotyphops braminus* (Daudin, 1803). Herpetozoa.

[76] Valdeón A, Perera A, Costa S, Sampaio F, Carretero MA (2010). Evidencia de una introducción de *Podarcis sicula* desde Italia a España asociada a una importación de olivos (*Olea europaea*). Boletín de la Asociación Herpetológica Española.

[77] Rivera X, Arribas O, Carranza S, Maluquer-Margalef J (2011). An introduction of *Podarcis sicula* in Catalonia (NE Iberian Peninsula) on imported olive trees. Butlletí de la Societat Catalana d’Herpetologia.

[78] González de la Vega J, González-García J, García-Pulido T, González-García G (2001). *Podarcis sicula* (Lagartija italiana), primera cita para Portugal. Boletín de la Asociación Herpetológica Española.

[79] Bruekers J (2006). Waarnemingen aan de Ruïnehagedis (*Podarcis sicula sicula*) en de Muurgekko (*Tarentola mauritanica*) in Noord-Italië (Gardameer). Lacerta.

[80] Delaugerre MJ, Holthof J (2014). The nursery trade: A stowaway gecko for a no return trip outside its natural range. Boletín de la Asociación Herpetológica Española.

[81] Galil BS, Nehring S, Panov V (2007). Waterways as invasion highways—Impact of climate change and globalization. Biol. Invasions.

[82] Rato C, Perera A, Carranza S, Harris DJ (2013). Evolutionary patterns of the mitochondrial genome in the Moorish gecko, *Tarentola mauritanica*. Gene.

[83] Harris DJ, Carretero MA, Corti C, Lo Cascio P (2009). Genetic affinities of *Tarentola mauritanica* (Reptilia: Gekkonidae) from Lampedusa and Conigli islet (SW Italy). North-Western J. Zool..

[84] Sambrook J, Fritsch EF, Maniatis T (1989). Molecular Cloning: A Laboratory Manual.

[85] Arranz SE (2013). Permanent genetic resources added to molecular ecology resources database 1 December 2012–31 January 2013. Mol. Ecol. Resour..

[86] Chatterji S, Pachter L (2006). Reference based annotation with GeneMapper. Genome Biol..

[87] van Oosterhout C, Hutchinson WF, Wills DPM, Shipley P (2004). MICRO-CHECKER: Software for identifying and correcting genotyping errors in microsatellite data. Mol. Ecol. Resour..

[88] Rousset F (2008). Genepop'007: A complete reimplementation of the Genepop software for Windows and Linux. Mol. Ecol. Resour..

[89] Raymond M, Rousset F (1995). GENEPOP (version 1.2): Population genetics software for exact tests and ecumenicism. J. Hered..

[90] Benjamini Y, Hochberg Y (1995). Controlling the false discovery rate: A practical and powerful approach to multiple testing. J. R. Stat. Soc. B.

[91] Strimmer K (2008). fdrtool: A versatile R package for estimating local and tail area-based false discovery rates. Bioinformatics.

[92] R Core Team. R: A language and environment for statistical computing. R Foundation for Statistical Computing, Vienna, Austria. https://www.R-project.org/ (2022).

[93] Keenan K, McGinnity P, Cross TF, Crozier WW, Prodöhl PA (2013). diveRsity: An R package for the estimation and exploration of population genetics parameters and their associated errors. Methods Ecol. Evol..

[94] Goudet J (2005). Hierfstat, a package for R to compute and test hierarchical F-statistics. Mol. Ecol. Notes.

[95] QGIS Geographic Information System. Open Source Geospatial Foundation Project [Computer software]. http://qgis.osgeo.org (2020).

[96] Weir BS, Cockerham CC (1984). Estimating F-statistics for the analysis of population structure. Evolution.

[97] Meirmans PG, Hedrick PW (2011). Assessing population structure: FST and related measures. Mol. Ecol. Resour..

[98] Pritchard JK, Stephens M, Donnelly P (2000). Inference of population structure using multilocus genotype data. Genetics.

[99] Falush D, Stephens M, Pritchard JK (2003). Inference of population structure using multilocus genotype data: Linked loci and correlated allele frequencies. Genetics.

[100] Earl DA, Holdt BM (2012). Structure harvester: A website and program for visualizing STRUCTURE output and implementing the Evanno method. Conserv. Genet. Resour..

[101] Kopelman NM, Mayzel J, Jakobsson M, Rosenberg NA, Mayrose I (2015). Clumpak: A program for identifying clustering modes and packaging population structure inferences across K. Mol. Ecol. Resour..

[102] Gerritsen, H. mapplots: Data Visualisation on Maps. R package version 1.5.1. https://CRAN.R-project.org/package=mapplots (2018).

[103] Caye K, Jay F, Michel O, Francois O (2018). Fast inference of individual admixture coefficients using geographic data. Ann. Appl. Stat..

[104] Jombart T, Devillard S, Balloux F (2010). Discriminant analysis of principal components: A new method for the analysis of genetically structured populations. BMC Genet..

[105] Chen C, Durand E, Forbes F, Francois O (2007). Bayesian clustering algorithms ascertaining spatial population structure: A new computer program and a comparison study. Mol. Ecol. Notes.

[106] Beerli P, Palczewski M (2010). Unified framework to evaluate panmixia and migration direction among multiple sampling locations. Genetics.

[107] Beerli, P. MIGRATE documentation (version 3.0). Technical Report. http://popgen.sc.fsu.edu (2008).

[108] Rambaut A, Drummond AJ, Xie D, Baele G, Suchard MA (2018). Posterior summarisation in Bayesian phylogenetics using Tracer 1.7. Syst. Biol..

[109] Gu Z, Gu L, Eils R, Schlesner M, Brors B (2014). *circlize* implements and enhances circular visualization in R. Bioinformatics.

