# Supplementary material for: Colonization routes uncovered in a widely introduced Mediterranean gecko, Tarentola mauritanica
Source: Sci Rep. 2023 Oct 4;13:16681. doi: 10.1038/s41598-023-43704-8 (PMC10551029; doi:10.1038/s41598-023-43704-8)
Supplement: Supplementary file 1 — Supplementary Information. [file 41598_2023_43704_MOESM1_ESM.docx]

**SUPPLEMENTARY MATERIAL**

**TABLES**

**Table S1.** Sampling information regarding all individuals of *Tarentola* *mauritanica* used in this study.

| **Specimen Code** | **Population Code** | **Locality** | **Province** | **Country** | **Source** |
| --- | --- | --- | --- | --- | --- |
| DB31155 | 3 | Bastia | Corsica | France | This study ** |
| DB31156 | 3 | Bastia | Corsica | France | This study ** |
| DB31157 | 3 | Bastia | Corsica | France | This study ** |
| DB31158 | 3 | Bastia | Corsica | France | This study ** |
| DB31159 | 3 | Bastia | Corsica | France | This study ** |
| DB31160 | 3 | Bastia | Corsica | France | This study ** |
| DB31161 | 4 | Cape Corse, Pietracorbara, l'Ornetu | Corsica | France | This study ** |
| DB31162 | 4 | Cape Corse, Pietracorbara, l'Ornetu | Corsica | France | This study ** |
| DB31163 | 4 | Cape Corse, Pietracorbara, l'Ornetu | Corsica | France | This study ** |
| DB31164 | 4 | Cape Corse, Pietracorbara, l'Ornetu | Corsica | France | This study ** |
| DB31165 | 4 | Cape Corse, Pietracorbara, l'Ornetu | Corsica | France | This study ** |
| DB31166 | 4 | Cape Corse, Pietracorbara, l'Ornetu | Corsica | France | This study ** |
| DB31167 | 4 | Cape Corse, Pietracorbara, l'Ornetu | Corsica | France | This study ** |
| DB31148 | 5 | Scandula, Elbu | Corsica | France | This study ** |
| DB31149 | 5 | Scandula, Elbu | Corsica | France | This study ** |
| DB31150 | 5 | Scandula, Elbu | Corsica | France | This study ** |
| DB31151 | 5 | Scandula, Elbu | Corsica | France | This study ** |
| DB31152 | 5 | Scandula, Elbu | Corsica | France | This study ** |
| DB31153 | 5 | Scandula, Elbu | Corsica | France | This study ** |
| DB31154 | 5 | Scandula, Elbu | Corsica | France | This study ** |
| DB31587 | 6 | Piolenc | Provence-Alpes-Côte d'Azur | France | This study ** |
| DB31588 | 6 | Piolenc | Provence-Alpes-Côte d'Azur | France | This study ** |
| DB31589 | 6 | Piolenc | Provence-Alpes-Côte d'Azur | France | This study ** |
| DB31590 | 6 | Piolenc | Provence-Alpes-Côte d'Azur | France | This study ** |
| DB31591 | 6 | Piolenc | Provence-Alpes-Côte d'Azur | France | This study ** |
| DB31573 | 7 | Montpellier, Avenue de la Justice | Occitanie | France | EPHE-CEFE-CNRS collection |
| DB31575 | 7 | Montpellier, UM2 | Occitanie | France | EPHE-CEFE-CNRS collection |
| DB31576 | 7 | Montpellier, 29 rue Figuerolles | Occitanie | France | EPHE-CEFE-CNRS collection |
| DB31579 | 7 | Montpellier, CEFE-CNRS, pièce de Paul Insenmann | Occitanie | France | EPHE-CEFE-CNRS collection |
| DB31580 | 7 | Montpellier, Cité universitaire de la Colombière | Occitanie | France | EPHE-CEFE-CNRS collection |
| DB31583 | 7 | Montpellier | Occitanie | France | EPHE-CEFE-CNRS collection |
| DB31585 | 7 | Montpellier, angle rue du Terral x rue de la Valfère | Occitanie | France | EPHE-CEFE-CNRS collection |
| DB31327 | 8 | Banyuls-sur-Mer | Occitanie | France | Koenig Museum collection |
| DB31328 | 8 | Banyuls-sur-Mer | Occitanie | France | Koenig Museum collection |
| DB31329 | 8 | between Argelès and Collioure | Occitanie | France | Koenig Museum collection |
| DB31330 | 8 | Argelês-sur-Mer | Occitanie | France | Koenig Museum collection |
| DB31331 | 8 | Banuyls-sur-Mer | Occitanie | France | Koenig Museum collection |
| DB31577 | 8 | Banyuls-sur-Mer | Occitanie | France | EPHE-CEFE-CNRS collection |
| DB173 | 9 | Crete | Crete | Greece | Previous studies * |
| DB174 | 9 | Crete | Crete | Greece | Previous studies * |
| DB2142 | 9 | Alikarnassos, SEAP,Irakleiou | Crete | Greece | Previous studies * |
| DB2150 | 9 | Kolokytha isl., Lasithiou | Crete | Greece | Previous studies * |
| DB2161 | 9 | Dia isl., Irakleiou | Crete | Greece | Previous studies * |
| DB2172 | 9 | Dia isl., Irakleiou | Crete | Greece | Previous studies * |
| DB14498 | 12 | Michliffen | Ifrane | Morocco | Previous studies * |
| DB14703 | 12 | Michliffen | Ifrane | Morocco | Previous studies * |
| DB14717 | 12 | Michliffen | Ifrane | Morocco | Previous studies * |
| DB14715 | 12 | Michliffen | Ifrane | Morocco | Previous studies * |
| DB14776 | 12 | Michliffen | Ifrane | Morocco | Previous studies * |
| DB15484 | 12 | Michliffen | Ifrane | Morocco | Previous studies * |
| DB15485 | 12 | Michliffen | Ifrane | Morocco | Previous studies * |
| DB15486 | 12 | Michliffen | Ifrane | Morocco | Previous studies * |
| DB15487 | 12 | Michliffen | Ifrane | Morocco | Previous studies * |
| DB15488 | 12 | Michliffen | Ifrane | Morocco | Previous studies * |
| DB15489 | 12 | Michliffen | Ifrane | Morocco | Previous studies * |
| DB15490 | 12 | Michliffen | Ifrane | Morocco | Previous studies * |
| DB15491 | 12 | Michliffen | Ifrane | Morocco | Previous studies * |
| DB25331 | 12 | Michliffen | Ifrane | Morocco | Previous studies * |
| DB25361 | 12 | Michliffen | Ifrane | Morocco | Previous studies * |
| DB25364 | 12 | Michliffen | Ifrane | Morocco | Previous studies * |
| DB23864 | 12 | 5km S of Azrou | Ifrane | Morocco | Previous studies * |
| DB23865 | 12 | 5km S of Azrou | Ifrane | Morocco | Previous studies * |
| DB2364 | 12 | 5km S of Azrou | Ifrane | Morocco | Previous studies * |
| DB23861 | 12 | Balcon D'Ito | Ifrane | Morocco | Previous studies * |
| DB2354 | 12 | 15km N of Azrou (Balcon d' Ito) | Ifrane | Morocco | Previous studies * |
| DB2355 | 12 | 15km N of Azrou (Balcon d' Ito) | Ifrane | Morocco | Previous studies * |
| DB2370 | 12 | 15km N of Azrou (Balcon d' Ito) | Ifrane | Morocco | Previous studies * |
| DB2372 | 12 | 15km N of Azrou (Balcon d' Ito) | Ifrane | Morocco | Previous studies * |
| DB310 | 12 | Michliffen | Michliffen | Morocco | Previous studies * |
| DB312 | 12 | Michliffen | Michliffen | Morocco | Previous studies * |
| DB14649 | 13 | Volubilis | Meknès | Morocco | Previous studies * |
| DB14650 | 13 | Volubilis | Meknès | Morocco | Previous studies * |
| DB75 | 13 | El Amma |  | Morocco | Previous studies * |
| DB940 | 13 | Défilé | Sidi Kacem | Morocco | Previous studies * |
| DB957 | 13 | Défilé | Sidi Kacem | Morocco | Previous studies * |
| DB19951 | 14 | Dlalha | Kénitra | Morocco | Previous studies * |
| DB19952 | 14 | Dlalha | Kénitra | Morocco | Previous studies * |
| DB322 | 14 | Moulay Bousselham | Moulay Bousselham | Morocco | Previous studies * |
| DB323 | 14 | Moulay Bousselham | Moulay Bousselham | Morocco | Previous studies * |
| DB324 | 14 | Moulay Bousselham | Moulay Bousselham | Morocco | Previous studies * |
| DB15582 | 15 | Ksar es Seghir | Fahs-Anjra | Morocco | Previous studies * |
| DB15583 | 15 | Ksar es Seghir | Fahs-Anjra | Morocco | Previous studies * |
| DB15584 | 15 | Eddalya | Tetouan | Morocco | Previous studies * |
| DB15585 | 15 | Eddalya | Tetouan | Morocco | Previous studies * |
| DB15586 | 15 | Eddalya | Tetouan | Morocco | Previous studies * |
| DB460 | 16 | Saida | Mouth of oued Moulouya | Morocco | Previous studies * |
| DB461 | 16 | Saida | Mouth of oued Moulouya | Morocco | Previous studies * |
| DB462 | 16 | Saida | Mouth of oued Moulouya | Morocco | Previous studies * |
| DB463 | 16 | Saida | Mouth of oued Moulouya | Morocco | Previous studies * |
| DB464 | 16 | Saida | Mouth of oued Moulouya | Morocco | Previous studies * |
| DB21240 | 17 | Évora (Mitra) | Évora | Portugal | Previous studies * |
| DB21263 | 17 | Évora (Mitra) | Évora | Portugal | Previous studies * |
| DB21264 | 17 | Évora (Mitra) | Évora | Portugal | Previous studies * |
| DB21269 | 17 | Évora (Mitra) | Évora | Portugal | Previous studies * |
| DB27505 | 17 | Évora | Évora | Portugal | Previous studies * |
| DB27506 | 17 | Évora | Évora | Portugal | Previous studies * |
| DB27507 | 17 | Évora | Évora | Portugal | Previous studies * |
| DB27508 | 17 | Évora | Évora | Portugal | Previous studies * |
| DB27509 | 17 | Évora | Évora | Portugal | Previous studies * |
| DB27510 | 17 | Évora | Évora | Portugal | Previous studies * |
| DB27511 | 17 | Évora | Évora | Portugal | Previous studies * |
| DB27512 | 17 | Évora | Évora | Portugal | Previous studies * |
| DB27513 | 17 | Évora | Évora | Portugal | Previous studies * |
| DB27514 | 17 | Évora | Évora | Portugal | Previous studies * |
| DB27515 | 17 | Évora | Évora | Portugal | Previous studies * |
| DB27516 | 17 | Évora | Évora | Portugal | Previous studies * |
| DB27521 | 17 | Évora | Évora | Portugal | Previous studies * |
| DB16792 | 17 | Évora cidade | Évora | Portugal | Previous studies * |
| DB20525 | 17 | Évora cidade | Évora | Portugal | Previous studies * |
| DB21260 | 17 | Évora cidade | Évora | Portugal | Previous studies * |
| DB21261 | 17 | Évora cidade | Évora | Portugal | Previous studies * |
| DB21262 | 17 | Évora cidade | Évora | Portugal | Previous studies * |
| DB21370 | 17 | Évora cidade | Évora | Portugal | Previous studies * |
| DB21374 | 17 | Évora cidade | Évora | Portugal | Previous studies * |
| DB21397 | 17 | Évora cidade | Évora | Portugal | Previous studies * |
| DB21399 | 17 | Évora cidade | Évora | Portugal | Previous studies * |
| DB21372 | 17 | Évora cidade | Évora | Portugal | Previous studies * |
| DB21382 | 17 | Évora cidade | Évora | Portugal | Previous studies * |
| DB21389 | 17 | Évora cidade | Évora | Portugal | Previous studies * |
| DB21376 | 17 | Évora cidade | Évora | Portugal | Previous studies * |
| DB21377 | 17 | Évora cidade | Évora | Portugal | Previous studies * |
| DB21379 | 17 | Évora cidade | Évora | Portugal | Previous studies * |
| DB21380 | 17 | Évora cidade | Évora | Portugal | Previous studies * |
| DB21384 | 17 | Évora cidade | Évora | Portugal | Previous studies * |
| DB21385 | 17 | Évora cidade | Évora | Portugal | Previous studies * |
| DB21386 | 17 | Évora cidade | Évora | Portugal | Previous studies * |
| DB21387 | 17 | Évora cidade | Évora | Portugal | Previous studies * |
| DB21388 | 17 | Évora cidade | Évora | Portugal | Previous studies * |
| DB21391 | 17 | Évora cidade | Évora | Portugal | Previous studies * |
| DB21392 | 17 | Évora cidade | Évora | Portugal | Previous studies * |
| DB21393 | 17 | Évora cidade | Évora | Portugal | Previous studies * |
| DB21395 | 17 | Évora cidade | Évora | Portugal | Previous studies * |
| DB21396 | 17 | Évora cidade | Évora | Portugal | Previous studies * |
| DB27949 | 18 | Tui, next to the old customs at the border with Portugal | Pontevedra | Portugal | Previous studies * |
| DB27951 | 18 | Tui, next to the old customs at the border with Portugal | Pontevedra | Portugal | Previous studies * |
| DB27952 | 18 | Tui, next to the old customs at the border with Portugal | Pontevedra | Portugal | Previous studies * |
| DB27957 | 18 | Tui, next to the old customs at the border with Portugal | Pontevedra | Portugal | Previous studies * |
| DB27963 | 18 | Tui, next to the old customs at the border with Portugal | Pontevedra | Portugal | Previous studies * |
| DB27976 | 18 | Tui, next to the old customs at the border with Portugal | Pontevedra | Portugal | Previous studies * |
| DB27978 | 18 | Tui, next to the old customs at the border with Portugal | Pontevedra | Portugal | Previous studies * |
| DB27985 | 18 | Tui, next to the old customs at the border with Portugal | Pontevedra | Portugal | Previous studies * |
| DB27986 | 18 | Tui, next to the old customs at the border with Portugal | Pontevedra | Portugal | Previous studies * |
| DB27987 | 18 | Tui, next to the old customs at the border with Portugal | Pontevedra | Portugal | Previous studies * |
| DB27953 | 19 | Quinta do Carriçal | Portimão | Portugal | Previous studies * |
| DB21241 | 19 | Quinta do Carriçal | Portimão | Portugal | Previous studies * |
| DB21242 | 19 | Quinta do Carriçal | Portimão | Portugal | Previous studies * |
| DB21243 | 19 | Quinta do Carriçal | Portimão | Portugal | Previous studies * |
| DB21244 | 19 | Quinta do Carriçal | Portimão | Portugal | Previous studies * |
| DB21245 | 19 | Quinta do Carriçal | Portimão | Portugal | Previous studies * |
| DB21246 | 19 | Quinta do Carriçal | Portimão | Portugal | Previous studies * |
| DB21247 | 19 | Quinta do Carriçal | Portimão | Portugal | Previous studies * |
| DB21248 | 19 | Quinta do Carriçal | Portimão | Portugal | Previous studies * |
| DB21249 | 19 | Quinta do Carriçal | Portimão | Portugal | Previous studies * |
| DB21250 | 19 | Quinta do Carriçal | Portimão | Portugal | Previous studies * |
| DB21251 | 19 | Quinta do Carriçal | Portimão | Portugal | Previous studies * |
| DB21252 | 19 | Quinta do Carriçal | Portimão | Portugal | Previous studies * |
| DB21253 | 19 | Quinta do Carriçal | Portimão | Portugal | Previous studies * |
| DB21254 | 19 | Quinta do Carriçal | Portimão | Portugal | Previous studies * |
| DB21255 | 19 | Quinta do Carriçal | Portimão | Portugal | Previous studies * |
| DB21256 | 19 | Quinta do Carriçal | Portimão | Portugal | Previous studies * |
| DB21257 | 19 | Quinta do Carriçal | Portimão | Portugal | Previous studies * |
| DB21258 | 19 | Quinta do Carriçal | Portimão | Portugal | Previous studies * |
| DB21265 | 19 | Quinta do Carriçal | Portimão | Portugal | Previous studies * |
| DB21266 | 19 | Quinta do Carriçal | Portimão | Portugal | Previous studies * |
| DB21267 | 19 | Quinta do Carriçal | Portimão | Portugal | Previous studies * |
| DB21268 | 19 | Quinta do Carriçal | Portimão | Portugal | Previous studies * |
| DB27517 | 19 | Serra de Mochique | Portimão | Portugal | Previous studies * |
| DB27518 | 19 | Serra de Mochique | Portimão | Portugal | Previous studies * |
| DB27519 | 19 | Serra de Mochique | Portimão | Portugal | Previous studies * |
| DB27520 | 19 | Serra de Mochique | Portimão | Portugal | Previous studies * |
| DB27522 | 19 | Serra de Mochique | Portimão | Portugal | Previous studies * |
| DB27523 | 19 | Serra de Mochique | Portimão | Portugal | Previous studies * |
| DB27524 | 19 | Serra de Mochique | Portimão | Portugal | Previous studies * |
| DB27525 | 19 | Serra de Mochique | Portimão | Portugal | Previous studies * |
| DB27526 | 19 | Serra de Mochique | Portimão | Portugal | Previous studies * |
| DB27527 | 19 | Serra de Mochique | Portimão | Portugal | Previous studies * |
| DB27528 | 19 | Serra de Mochique | Portimão | Portugal | Previous studies * |
| DB27529 | 19 | Serra de Mochique | Portimão | Portugal | Previous studies * |
| DB27530 | 19 | Serra de Mochique | Portimão | Portugal | Previous studies * |
| DB27531 | 19 | Serra de Mochique | Portimão | Portugal | Previous studies * |
| DB27532 | 19 | Serra de Mochique | Portimão | Portugal | Previous studies * |
| DB27533 | 19 | Serra de Mochique | Portimão | Portugal | Previous studies * |
| DB27534 | 19 | Serra de Mochique | Portimão | Portugal | Previous studies * |
| DB27535 | 19 | Serra de Mochique | Portimão | Portugal | Previous studies * |
| DB27536 | 19 | Serra de Mochique | Portimão | Portugal | Previous studies * |
| DB27538 | 19 | Serra de Mochique | Portimão | Portugal | Previous studies * |
| DB25644 | 19 | Serra de Mochique | Portimão | Portugal | Previous studies * |
| DB27950 | 19 | Serra de Mochique | Portimão | Portugal | Previous studies * |
| DB27954 | 19 | Serra de Mochique | Portimão | Portugal | Previous studies * |
| DB27961 | 19 | Serra de Mochique | Portimão | Portugal | Previous studies * |
| DB27965 | 19 | Serra de Mochique | Portimão | Portugal | Previous studies * |
| DB27967 | 19 | Serra de Mochique | Portimão | Portugal | Previous studies * |
| DB27969 | 19 | Serra de Mochique | Portimão | Portugal | Previous studies * |
| DB27970 | 19 | Serra de Mochique | Portimão | Portugal | Previous studies * |
| DB27972 | 19 | Serra de Mochique | Portimão | Portugal | Previous studies * |
| DB27973 | 19 | Serra de Mochique | Portimão | Portugal | Previous studies * |
| DB27974 | 19 | Serra de Mochique | Portimão | Portugal | Previous studies * |
| DB27975 | 19 | Serra de Mochique | Portimão | Portugal | Previous studies * |
| DB27981 | 19 | Serra de Mochique | Portimão | Portugal | Previous studies * |
| DB27982 | 19 | Serra de Mochique | Portimão | Portugal | Previous studies * |
| DB27983 | 19 | Serra de Mochique | Portimão | Portugal | Previous studies * |
| DB27989 | 19 | Serra de Mochique | Portimão | Portugal | Previous studies * |
| DB27990 | 19 | Serra de Mochique | Portimão | Portugal | Previous studies * |
| DB31333 | 20 | Estartit: Islas Medas | Catalunya | Spain | Koenig Museum collection |
| DB31334 | 20 | Estartit: Islas Medas | Catalunya | Spain | Koenig Museum collection |
| DB31335 | 20 | Estartit: Islas Medas | Catalunya | Spain | Koenig Museum collection |
| DB31336 | 20 | Estartit: Islas Medas | Catalunya | Spain | Koenig Museum collection |
| DB31337 | 20 | Estartit: Islas Medas | Catalunya | Spain | Koenig Museum collection |
| DB31338 | 20 | Estartit: Islas Medas | Catalunya | Spain | Koenig Museum collection |
| DB31339 | 20 | Estartit: Islas Medas | Catalunya | Spain | Koenig Museum collection |
| DB31340 | 20 | Estartit: Islas Medas | Catalunya | Spain | Koenig Museum collection |
| DB31341 | 20 | Estartit: Islas Medas | Catalunya | Spain | Koenig Museum collection |
| DB31342 | 20 | Estartit: Islas Medas | Catalunya | Spain | Koenig Museum collection |
| DB9327 | 21 | Platja Gran | Menorca | Spain | Previous studies * |
| DB9213 | 21 | S'Hostal quarries, Lithica | Menorca | Spain | Previous studies * |
| DB9222 | 21 | S'Hostal quarries, Lithica | Menorca | Spain | Previous studies * |
| DB9226 | 21 | S'Hostal quarries, Lithica | Menorca | Spain | Previous studies * |
| DB9235 | 21 | S'Hostal quarries, Lithica | Menorca | Spain | Previous studies * |
| DB9292 | 21 | S'Hostal quarries, Lithica | Menorca | Spain | Previous studies * |
| DB9296 | 21 | S'Hostal quarries, Lithica | Menorca | Spain | Previous studies * |
| DB9302 | 21 | S'Hostal quarries, Lithica | Menorca | Spain | Previous studies * |
| DB208 | 21 | Lithica | Menorca | Spain | Previous studies * |
| DB209 | 21 | Lithica | Menorca | Spain | Previous studies * |
| DB395 | 22 | Matalascanas | Huelva | Spain | Previous studies * |
| DB21550 | 22 | Matalascañas | Huelva | Spain | Previous studies * |
| DB22070 | 22 | Matalascañas | Huelva | Spain | Previous studies * |
| DB22072 | 22 | Matalascañas | Huelva | Spain | Previous studies * |
| DB22086 | 22 | Matalascañas | Huelva | Spain | Previous studies * |
| DB22087 | 22 | Matalascañas | Huelva | Spain | Previous studies * |
| DB22088 | 22 | Matalascañas | Huelva | Spain | Previous studies * |
| DB22089 | 22 | Matalascañas | Huelva | Spain | Previous studies * |
| DB22090 | 22 | Matalascañas | Huelva | Spain | Previous studies * |
| DB22091 | 22 | Matalascañas | Huelva | Spain | Previous studies * |
| DB22092 | 22 | Matalascañas | Huelva | Spain | Previous studies * |
| DB22093 | 22 | Matalascañas | Huelva | Spain | Previous studies * |
| DB22094 | 22 | Matalascañas | Huelva | Spain | Previous studies * |
| DB22095 | 22 | Matalascañas | Huelva | Spain | Previous studies * |
| DB22096 | 22 | Matalascañas | Huelva | Spain | Previous studies * |
| DB22097 | 22 | Matalascañas | Huelva | Spain | Previous studies * |
| DB22098 | 22 | Matalascañas | Huelva | Spain | Previous studies * |
| DB22099 | 22 | Matalascañas | Huelva | Spain | Previous studies * |
| DB22100 | 22 | Matalascañas | Huelva | Spain | Previous studies * |
| DB22101 | 22 | Matalascañas | Huelva | Spain | Previous studies * |
| DB22102 | 22 | Matalascañas | Huelva | Spain | Previous studies * |
| DB22103 | 22 | Matalascañas | Huelva | Spain | Previous studies * |
| DB22104 | 22 | Matalascañas | Huelva | Spain | Previous studies * |
| DB22105 | 22 | Matalascañas | Huelva | Spain | Previous studies * |
| DB22106 | 22 | Matalascañas | Huelva | Spain | Previous studies * |
| DB22107 | 22 | Matalascañas | Huelva | Spain | Previous studies * |
| DB22108 | 22 | Matalascañas | Huelva | Spain | Previous studies * |
| DB22109 | 22 | Matalascañas | Huelva | Spain | Previous studies * |
| DB22110 | 22 | Matalascañas | Huelva | Spain | Previous studies * |
| DB22111 | 22 | Matalascañas | Huelva | Spain | Previous studies * |
| DB22112 | 22 | Matalascañas | Huelva | Spain | Previous studies * |
| DB22113 | 22 | Matalascañas | Huelva | Spain | Previous studies * |
| DB279 | 22 | Matalascañas | Huelva | Spain | Previous studies * |
| DB21590 | 23 | Palacio de Doñana | Huelva | Spain | Previous studies * |
| DB21591 | 23 | Palacio de Doñana | Huelva | Spain | Previous studies * |
| DB21592 | 23 | Palacio de Doñana | Huelva | Spain | Previous studies * |
| DB21593 | 23 | Palacio de Doñana | Huelva | Spain | Previous studies * |
| DB21594 | 23 | Palacio de Doñana | Huelva | Spain | Previous studies * |
| DB21595 | 23 | Palacio de Doñana | Huelva | Spain | Previous studies * |
| DB21596 | 23 | Palacio de Doñana | Huelva | Spain | Previous studies * |
| DB21597 | 23 | Palacio de Doñana | Huelva | Spain | Previous studies * |
| DB21598 | 23 | Palacio de Doñana | Huelva | Spain | Previous studies * |
| DB21994 | 23 | Palacio de Doñana | Huelva | Spain | Previous studies * |
| DB21995 | 23 | Palacio de Doñana | Huelva | Spain | Previous studies * |
| DB21996 | 23 | Palacio de Doñana | Huelva | Spain | Previous studies * |
| DB21997 | 23 | Palacio de Doñana | Huelva | Spain | Previous studies * |
| DB21998 | 23 | Palacio de Doñana | Huelva | Spain | Previous studies * |
| DB21999 | 23 | Palacio de Doñana | Huelva | Spain | Previous studies * |
| DB22000 | 23 | Palacio de Doñana | Huelva | Spain | Previous studies * |
| DB22001 | 23 | Palacio de Doñana | Huelva | Spain | Previous studies * |
| DB22002 | 23 | Palacio de Doñana | Huelva | Spain | Previous studies * |
| DB22003 | 23 | Palacio de Doñana | Huelva | Spain | Previous studies * |
| DB22004 | 23 | Palacio de Doñana | Huelva | Spain | Previous studies * |
| DB22005 | 23 | Palacio de Doñana | Huelva | Spain | Previous studies * |
| DB22006 | 23 | Palacio de Doñana | Huelva | Spain | Previous studies * |
| DB22007 | 23 | Palacio de Doñana | Huelva | Spain | Previous studies * |
| DB22008 | 23 | Palacio de Doñana | Huelva | Spain | Previous studies * |
| DB22009 | 23 | Palacio de Doñana | Huelva | Spain | Previous studies * |
| DB22010 | 23 | Palacio de Doñana | Huelva | Spain | Previous studies * |
| DB22011 | 23 | Palacio de Doñana | Huelva | Spain | Previous studies * |
| DB22012 | 23 | Palacio de Doñana | Huelva | Spain | Previous studies * |
| DB22013 | 23 | Palacio de Doñana | Huelva | Spain | Previous studies * |
| DB22014 | 23 | Palacio de Doñana | Huelva | Spain | Previous studies * |
| DB22015 | 23 | Palacio de Doñana | Huelva | Spain | Previous studies * |
| DB22016 | 23 | Palacio de Doñana | Huelva | Spain | Previous studies * |
| DB22017 | 23 | Palacio de Doñana | Huelva | Spain | Previous studies * |
| DB22018 | 23 | Palacio de Doñana | Huelva | Spain | Previous studies * |
| DB22019 | 23 | Palacio de Doñana | Huelva | Spain | Previous studies * |
| DB22020 | 23 | Palacio de Doñana | Huelva | Spain | Previous studies * |
| DB22021 | 23 | Palacio de Doñana | Huelva | Spain | Previous studies * |
| DB22022 | 23 | Palacio de Doñana | Huelva | Spain | Previous studies * |
| DB22023 | 23 | Palacio de Doñana | Huelva | Spain | Previous studies * |
| DB22024 | 23 | Palacio de Doñana | Huelva | Spain | Previous studies * |
| DB22025 | 23 | Palacio de Doñana | Huelva | Spain | Previous studies * |
| DB22026 | 23 | Palacio de Doñana | Huelva | Spain | Previous studies * |
| DB22027 | 23 | Palacio de Doñana | Huelva | Spain | Previous studies * |
| DB22028 | 23 | Palacio de Doñana | Huelva | Spain | Previous studies * |
| DB22029 | 23 | Palacio de Doñana | Huelva | Spain | Previous studies * |
| DB22071 | 23 | Palacio de Doñana | Huelva | Spain | Previous studies * |
| DB22073 | 23 | Palacio de Doñana | Huelva | Spain | Previous studies * |
| DB394 | 23 | Palacio de Doñana | Huelva | Spain | Previous studies * |
| DB31332 | 24 | Barcelona, surroundings | Catalunya | Spain | Koenig Museum collection |
| DB31643 | 24 | Barcelona | Catalunya | Spain | Previous studies * |
| DB31644 | 24 | Barcelona | Catalunya | Spain | Previous studies * |
| DB31645 | 24 | Barcelona | Catalunya | Spain | Previous studies * |
| DB31646 | 24 | Barcelona | Catalunya | Spain | Previous studies * |
| DB31647 | 24 | Barcelona | Catalunya | Spain | Previous studies * |
| DB31648 | 24 | Barcelona | Catalunya | Spain | Previous studies * |
| DB31649 | 24 | Barcelona | Catalunya | Spain | Previous studies * |
| DB2352 | 25 | Chiclana de La Frontera | Cádiz | Spain | Previous studies * |
| DB155 | 25 | Barrosa | Cádiz | Spain | Previous studies * |
| DB308 | 25 | Chiclana de la Frontera | Barrosa | Spain | Previous studies * |
| DB365 | 25 | Barrosa | Cádiz | Spain | Previous studies * |
| DB369 | 25 | Barrosa | Cádiz | Spain | Previous studies * |
| DB376 | 25 | Chiclana de la Frontera | Cádiz | Spain | Previous studies * |
| DB1669 | 25 | Chiclana de la Frontera | Cádiz | Spain | Previous studies * |
| DB10558 | 27 | near Portocolom | Mallorca | Spain | Previous studies * |
| DB10576 | 27 | near Portocolom | Mallorca | Spain | Previous studies * |
| DB10598 | 27 | near Portocolom | Mallorca | Spain | Previous studies * |
| DB10318 | 27 | Portocolom | Mallorca | Spain | Previous studies * |
| DB10399 | 27 | Portocolom | Mallorca | Spain | Previous studies * |
| DB10456 | 27 | Portocolom | Mallorca | Spain | Previous studies * |
| DB10520 | 27 | Portocolom | Mallorca | Spain | Previous studies * |
| DB10552 | 27 | Portocolom | Mallorca | Spain | Previous studies * |
| DB31574 | 28 | transect 176 | Catalunya | Spain | EPHE-CEFE-CNRS collection |
| DB31581 | 28 | transect 176 | Catalunya | Spain | EPHE-CEFE-CNRS collection |
| DB31582 | 28 | transect 176 | Catalunya | Spain | EPHE-CEFE-CNRS collection |
| DB31584 | 28 | transect 176 | Catalunya | Spain | EPHE-CEFE-CNRS collection |
| DB31586 | 28 | transect 176 | Catalunya | Spain | EPHE-CEFE-CNRS collection |
| DB31140 | 29 | Galite island | Bizerte | Tunisia | This study ** |
| DB31141 | 29 | Galite island | Bizerte | Tunisia | This study ** |
| DB31142 | 29 | Galite island | Bizerte | Tunisia | This study ** |
| DB31143 | 29 | Galite island | Bizerte | Tunisia | This study ** |
| DB31144 | 29 | Galite island | Bizerte | Tunisia | This study ** |
| DB31145 | 29 | Galite island | Bizerte | Tunisia | This study ** |
| DB31146 | 29 | Galite island | Bizerte | Tunisia | This study ** |
| DB31147 | 29 | Galite island | Bizerte | Tunisia | This study ** |
| DB162 | 30 | Kebir | Kebir | Tunisia | Previous studies * |
| DB163 | 30 | Tabarka | Tabarka | Tunisia | Previous studies * |
| DB214 | 30 | Guelta Safra | Guelta Safra | Tunisia | Previous studies * |
| DB215 | 30 | Guelta Safra | Guelta Safra | Tunisia | Previous studies * |
| DB216 | 30 | Guelta Safra | Guelta Safra | Tunisia | Previous studies * |
| DB217 | 30 | Guelta Safra | Guelta Safra | Tunisia | Previous studies * |
| DB218 | 30 | Guelta Safra | Guelta Safra | Tunisia | Previous studies * |
| DB219 | 30 | Guelta Safra | Guelta Safra | Tunisia | Previous studies * |
| DB425 | 30 | Tabarka |  | Tunisia | Previous studies * |
| DB31592 | 31 | Forna, Eze | Alpes-Maritimes | France | This study ** |
| DB31593 | 31 | Forna, Eze | Alpes-Maritimes | France | This study ** |
| DB31594 | 31 | Forna, Eze | Alpes-Maritimes | France | This study ** |
| DB31595 | 31 | Forna, Eze | Alpes-Maritimes | France | This study ** |
| DB31596 | 31 | Forna, Eze | Alpes-Maritimes | France | This study ** |
| DB31597 | 31 | Forna, Eze | Alpes-Maritimes | France | This study ** |
| DB31598 | 31 | Forna, Eze | Alpes-Maritimes | France | This study ** |
| DB31599 | 31 | Forna, Eze | Alpes-Maritimes | France | This study ** |
| DB31600 | 31 | Forna, Eze | Alpes-Maritimes | France | This study ** |
| DB31601 | 31 | Forna, Eze | Alpes-Maritimes | France | This study ** |
| DB31602 | 32 | Quartier Endoume island | Marseille | France | This study ** |
| DB31603 | 32 | Quartier Endoume island | Marseille | France | This study ** |
| DB31604 | 32 | Quartier Endoume island | Marseille | France | This study ** |
| DB31605 | 32 | Quartier Endoume island | Marseille | France | This study ** |
| DB31606 | 32 | Quartier Endoume island | Marseille | France | This study ** |
| DB31607 | 32 | Quartier Endoume island | Marseille | France | This study ** |
| DB31608 | 32 | Quartier Endoume island | Marseille | France | This study ** |
| DB31609 | 32 | Quartier Endoume island | Marseille | France | This study ** |
| DB31610 | 32 | Quartier Endoume island | Marseille | France | This study ** |
| DB31611 | 33 | St Marguerite island | Cannes | France | This study ** |
| DB31612 | 33 | St Marguerite island | Cannes | France | This study ** |
| DB31613 | 33 | St Marguerite island | Cannes | France | This study ** |
| DB31614 | 33 | St Marguerite island | Cannes | France | This study ** |
| DB31615 | 33 | St Marguerite island | Cannes | France | This study ** |
| DB31616 | 33 | St Marguerite island | Cannes | France | This study ** |
| DB31617 | 33 | St Marguerite island | Cannes | France | This study ** |
| DB31459 | 34 | Caniço (Garajau) | Madeira | Portugal | Previous studies * |
| DB31457 | 34 | Caniço (Garajau) | Madeira | Portugal | Previous studies * |
| DB31458 | 34 | Caniço (Garajau) | Madeira | Portugal | Previous studies * |
| DB31471 | 34 | Caniço (Garajau) | Madeira | Portugal | Previous studies * |
| DB31470 | 34 | Caniço (Garajau) | Madeira | Portugal | Previous studies * |
| DB31469 | 34 | Caniço (Garajau) | Madeira | Portugal | Previous studies * |
| DB31479 | 34 | Caniço (Garajau) | Madeira | Portugal | Previous studies * |
| DB31472 | 34 | Caniço (Garajau) | Madeira | Portugal | Previous studies * |
| DB31462 | 34 | Caniço (Garajau) | Madeira | Portugal | Previous studies * |
| DB31446 | 34 | Caniço (Garajau) | Madeira | Portugal | Previous studies * |
| DB31476 | 35 | Ribeira Brava | Madeira | Portugal | Previous studies * |
| DB31477 | 35 | Ribeira Brava | Madeira | Portugal | Previous studies * |
| DB31478 | 35 | Ribeira Brava | Madeira | Portugal | Previous studies * |
| DB31475 | 35 | Ribeira Brava | Madeira | Portugal | Previous studies * |
| DB31465 | 35 | Ribeira Brava | Madeira | Portugal | Previous studies * |
| DB31481 | 35 | Ribeira Brava | Madeira | Portugal | Previous studies * |
| DB31482 | 35 | Ribeira Brava | Madeira | Portugal | Previous studies * |
| DB31483 | 35 | Ribeira Brava | Madeira | Portugal | Previous studies * |
| DB31484 | 35 | Ribeira Brava | Madeira | Portugal | Previous studies * |
| DB31485 | 35 | Ribeira Brava | Madeira | Portugal | Previous studies * |
| DB31500 | 36 | Câmara de Lobos | Madeira | Portugal | Previous studies * |
| DB31502 | 36 | Câmara de Lobos | Madeira | Portugal | Previous studies * |
| DB31503 | 36 | Câmara de Lobos | Madeira | Portugal | Previous studies * |
| DB31504 | 36 | Câmara de Lobos | Madeira | Portugal | Previous studies * |
| DB31505 | 36 | Câmara de Lobos | Madeira | Portugal | Previous studies * |
| DB31506 | 36 | Câmara de Lobos | Madeira | Portugal | Previous studies * |
| DB31507 | 36 | Câmara de Lobos | Madeira | Portugal | Previous studies * |
| DB31508 | 36 | Câmara de Lobos | Madeira | Portugal | Previous studies * |
| DB31509 | 36 | Câmara de Lobos | Madeira | Portugal | Previous studies * |
| DB31510 | 36 | Câmara de Lobos | Madeira | Portugal | Previous studies * |
| DB31359 | 37 | Formosa Beach | Madeira | Portugal | Previous studies * |
| DB31360 | 37 | Formosa Beach | Madeira | Portugal | Previous studies * |
| DB31361 | 37 | Formosa Beach | Madeira | Portugal | Previous studies * |
| DB31422 | 37 | Formosa Beach | Madeira | Portugal | Previous studies * |
| DB31349 | 37 | Formosa Beach | Madeira | Portugal | Previous studies * |
| DB31354 | 37 | Formosa Beach | Madeira | Portugal | Previous studies * |
| DB31412 | 37 | Formosa Beach | Madeira | Portugal | Previous studies * |
| DB31351 | 37 | Formosa Beach | Madeira | Portugal | Previous studies * |
| DB31353 | 37 | Formosa Beach | Madeira | Portugal | Previous studies * |
| DB31355 | 37 | Formosa Beach | Madeira | Portugal | Previous studies * |
| DB31381 | 38 | Caniçal | Madeira | Portugal | Previous studies * |
| DB31383 | 38 | Caniçal | Madeira | Portugal | Previous studies * |
| DB31385 | 38 | Caniçal | Madeira | Portugal | Previous studies * |
| DB31386 | 38 | Caniçal | Madeira | Portugal | Previous studies * |
| DB31387 | 38 | Caniçal | Madeira | Portugal | Previous studies * |
| DB31530 | 38 | Caniçal | Madeira | Portugal | Previous studies * |
| DB31396 | 38 | Caniçal | Madeira | Portugal | Previous studies * |
| DB31393 | 38 | Caniçal | Madeira | Portugal | Previous studies * |
| DB31398 | 38 | Caniçal | Madeira | Portugal | Previous studies * |
| DB31379 | 38 | Caniçal | Madeira | Portugal | Previous studies * |
| DB31542 | 39 | Funchal | Madeira | Portugal | Previous studies * |
| DB31352 | 39 | Funchal | Madeira | Portugal | Previous studies * |
| DB31362 | 39 | Funchal | Madeira | Portugal | Previous studies * |
| DB31382 | 39 | Funchal | Madeira | Portugal | Previous studies * |
| DB31356 | 39 | Funchal | Madeira | Portugal | Previous studies * |
| DB31358 | 39 | Funchal | Madeira | Portugal | Previous studies * |
| DB31409 | 39 | Funchal | Madeira | Portugal | Previous studies * |
| DB31384 | 39 | Funchal | Madeira | Portugal | Previous studies * |
| DB31406 | 39 | Funchal | Madeira | Portugal | Previous studies * |
| DB31343 | 39 | Funchal | Madeira | Portugal | Previous studies * |
| DB31629 | 40 | Pennes-Mirabeau | Provence-Alpes-Côte d'Azur | France | This study ** |
| DB31630 | 40 | Pennes-Mirabeau | Provence-Alpes-Côte d'Azur | France | This study ** |
| DB31631 | 40 | Pennes-Mirabeau | Provence-Alpes-Côte d'Azur | France | This study ** |
| DB31632 | 40 | Pennes-Mirabeau | Provence-Alpes-Côte d'Azur | France | This study ** |
| DB31633 | 40 | Pennes-Mirabeau | Provence-Alpes-Côte d'Azur | France | This study ** |
| DB31634 | 40 | Pennes-Mirabeau | Provence-Alpes-Côte d'Azur | France | This study ** |
| DB31635 | 41 | Cascastel des Corbières | Occitanie | France | This study ** |
| DB31636 | 41 | Cascastel des Corbières | Occitanie | France | This study ** |
| DB31637 | 41 | Cascastel des Corbières | Occitanie | France | This study ** |
| DB31638 | 41 | Cascastel des Corbières | Occitanie | France | This study ** |
| DB31639 | 41 | Cascastel des Corbières | Occitanie | France | This study ** |
| AB1171 | 42 | Genova | Rapallo | Italy | Belluardo, et al. ^1^ |
| AB1170 | 42 | Genova | Rapallo | Italy | Belluardo *et al.* (2023) |
| AB1168 | 42 | Genova | Rapallo | Italy | Belluardo *et al.* (2023) |
| AB1155 | 42 | Genova | Rapallo | Italy | Belluardo *et al.* (2023) |
| AB1161 | 42 | Genova | Rapallo | Italy | Belluardo *et al.* (2023) |
| AB1158 | 42 | Genova | Rapallo | Italy | Belluardo *et al.* (2023) |
| AB1157 | 42 | Genova | Rapallo | Italy | Belluardo *et al.* (2023) |
| AB1052 | 42 | Genova | Rapallo | Italy | Belluardo *et al.* (2023) |
| AB1162 | 42 | Genova | Rapallo | Italy | Belluardo *et al.* (2023) |
| AB1160 | 42 | Genova | Rapallo | Italy | Belluardo *et al.* (2023) |
| AB1154 | 42 | Genova | Rapallo | Italy | Belluardo *et al.* (2023) |
| AB1217 | 42 | Genova | Rapallo | Italy | Belluardo *et al.* (2023) |
| AB1159 | 42 | Genova | Rapallo | Italy | Belluardo *et al.* (2023) |
| AB1172 | 42 | Genova | Rapallo | Italy | Belluardo *et al.* (2023) |
| AB1547 | 43 | Pisa | Calci | Italy | Belluardo *et al.* (2023) |
| AB1594 | 43 | Pisa | Calci | Italy | Belluardo *et al.* (2023) |
| AB1548 | 43 | Pisa | Calci | Italy | Belluardo *et al.* (2023) |
| AB1554 | 43 | Pisa | Calci | Italy | Belluardo *et al.* (2023) |
| AB1595 | 43 | Pisa | Calci | Italy | Belluardo *et al.* (2023) |
| AB1555 | 43 | Pisa | Calci | Italy | Belluardo *et al.* (2023) |
| AB1550 | 43 | Pisa | Calci | Italy | Belluardo *et al.* (2023) |
| AB1553 | 43 | Pisa | Calci | Italy | Belluardo *et al.* (2023) |
| AB1549 | 43 | Pisa | Calci | Italy | Belluardo *et al.* (2023) |
| AB1546 | 43 | Pisa | Calci | Italy | Belluardo *et al.* (2023) |
| AB1615 | 43 | Pisa | Calci | Italy | Belluardo *et al.* (2023) |
| AB1601 | 43 | Pisa | Calci | Italy | Belluardo *et al.* (2023) |
| AB1608 | 43 | Pisa | Calci | Italy | Belluardo *et al.* (2023) |
| AB1606 | 43 | Pisa | Calci | Italy | Belluardo *et al.* (2023) |
| AB1604 | 43 | Pisa | Calci | Italy | Belluardo *et al.* (2023) |
| AB1614 | 43 | Pisa | Calci | Italy | Belluardo *et al.* (2023) |
| AB1303 | 44 | Ascoli-Piceno | Ascoli, porto | Italy | Belluardo *et al.* (2023) |
| AB1300 | 44 | Ascoli-Piceno | Ascoli, porto | Italy | Belluardo *et al.* (2023) |
| AB1314 | 44 | Ascoli-Piceno | Ascoli, porto | Italy | Belluardo *et al.* (2023) |
| AB1316 | 44 | Ascoli-Piceno | Ascoli, porto | Italy | Belluardo *et al.* (2023) |
| AB1321 | 44 | Ascoli-Piceno | Ascoli, porto | Italy | Belluardo *et al.* (2023) |
| AB1313 | 44 | Ascoli-Piceno | Ascoli, porto | Italy | Belluardo *et al.* (2023) |
| AB1318 | 44 | Ascoli-Piceno | Ascoli, porto | Italy | Belluardo *et al.* (2023) |
| AB1322 | 44 | Ascoli-Piceno | Ascoli, porto | Italy | Belluardo *et al.* (2023) |
| AB1320 | 44 | Ascoli-Piceno | Ascoli, porto | Italy | Belluardo *et al.* (2023) |
| AB1325 | 44 | Ascoli-Piceno | Ascoli, porto | Italy | Belluardo *et al.* (2023) |
| AB2508 | 45 | Viterbo | Tarquinia | Italy | Belluardo *et al.* (2023) |
| AB2513 | 45 | Viterbo | Tarquinia | Italy | Belluardo *et al.* (2023) |
| AB2534 | 45 | Viterbo | Tarquinia | Italy | Belluardo *et al.* (2023) |
| AB2542 | 45 | Viterbo | Tarquinia | Italy | Belluardo *et al.* (2023) |
| AB2546 | 45 | Viterbo | Tarquinia | Italy | Belluardo *et al.* (2023) |
| AB2553 | 45 | Viterbo | Tarquinia | Italy | Belluardo *et al.* (2023) |
| AB2554 | 45 | Viterbo | Tarquinia | Italy | Belluardo *et al.* (2023) |
| AB2555 | 45 | Viterbo | Tarquinia | Italy | Belluardo *et al.* (2023) |
| AB2556 | 45 | Viterbo | Tarquinia | Italy | Belluardo *et al.* (2023) |
| AB2557 | 45 | Viterbo | Tarquinia | Italy | Belluardo *et al.* (2023) |
| AB2558 | 45 | Viterbo | Tarquinia | Italy | Belluardo *et al.* (2023) |
| AB2559 | 45 | Viterbo | Tarquinia | Italy | Belluardo *et al.* (2023) |
| AB2560 | 45 | Viterbo | Tarquinia | Italy | Belluardo *et al.* (2023) |
| AB2561 | 45 | Viterbo | Tarquinia | Italy | Belluardo *et al.* (2023) |
| AB2562 | 45 | Viterbo | Tarquinia | Italy | Belluardo *et al.* (2023) |
| AB2563 | 45 | Viterbo | Tarquinia | Italy | Belluardo *et al.* (2023) |
| AB2564 | 45 | Viterbo | Tarquinia | Italy | Belluardo *et al.* (2023) |
| AB2565 | 45 | Viterbo | Tarquinia | Italy | Belluardo *et al.* (2023) |
| AB2566 | 45 | Viterbo | Tarquinia | Italy | Belluardo *et al.* (2023) |
| AB2567 | 45 | Viterbo | Tarquinia | Italy | Belluardo *et al.* (2023) |
| AB1367 | 46 | Latina | Borgo Montello | Italy | Belluardo *et al.* (2023) |
| AB1360 | 46 | Latina | Borgo Montello | Italy | Belluardo *et al.* (2023) |
| AB1340 | 46 | Latina | Borgo Montello | Italy | Belluardo *et al.* (2023) |
| AB1337 | 46 | Latina | Borgo Montello | Italy | Belluardo *et al.* (2023) |
| AB1339 | 46 | Latina | Borgo Montello | Italy | Belluardo *et al.* (2023) |
| AB2568 | 46 | Latina | Borgo Montello | Italy | Belluardo *et al.* (2023) |
| AB2569 | 46 | Latina | Borgo Montello | Italy | Belluardo *et al.* (2023) |
| AB2570 | 46 | Latina | Borgo Montello | Italy | Belluardo *et al.* (2023) |
| AB2571 | 46 | Latina | Borgo Montello | Italy | Belluardo *et al.* (2023) |
| AB2572 | 46 | Latina | Borgo Montello | Italy | Belluardo *et al.* (2023) |
| AB2573 | 46 | Latina | Borgo Montello | Italy | Belluardo *et al.* (2023) |
| AB2574 | 46 | Latina | Borgo Montello | Italy | Belluardo *et al.* (2023) |
| AB2527 | 47 | Bari | Molfetta | Italy | Belluardo *et al.* (2023) |
| AB2535 | 47 | Bari | Molfetta | Italy | Belluardo *et al.* (2023) |
| AB2536 | 47 | Bari | Molfetta | Italy | Belluardo *et al.* (2023) |
| AB2537 | 47 | Bari | Molfetta | Italy | Belluardo *et al.* (2023) |
| AB2539 | 47 | Bari | Molfetta | Italy | Belluardo *et al.* (2023) |
| AB2540 | 47 | Bari | Molfetta | Italy | Belluardo *et al.* (2023) |
| AB2541 | 47 | Bari | Molfetta | Italy | Belluardo *et al.* (2023) |
| AB2543 | 47 | Bari | Molfetta | Italy | Belluardo *et al.* (2023) |
| AB2545 | 47 | Bari | Molfetta | Italy | Belluardo *et al.* (2023) |
| AB2552 | 47 | Bari | Molfetta | Italy | Belluardo *et al.* (2023) |
| AB1460 | 48 | Lecce | Vernole | Italy | Belluardo *et al.* (2023) |
| AB1480 | 48 | Lecce | Vernole | Italy | Belluardo *et al.* (2023) |
| AB1447 | 48 | Lecce | Vernole | Italy | Belluardo *et al.* (2023) |
| AB1443 | 48 | Lecce | Vernole | Italy | Belluardo *et al.* (2023) |
| AB1462 | 48 | Lecce | Vernole | Italy | Belluardo *et al.* (2023) |
| AB1528 | 48 | Lecce | Vernole | Italy | Belluardo *et al.* (2023) |
| AB1529 | 48 | Lecce | Vernole | Italy | Belluardo *et al.* (2023) |
| AB1530 | 48 | Lecce | Vernole | Italy | Belluardo *et al.* (2023) |
| AB1531 | 48 | Lecce | Vernole | Italy | Belluardo *et al.* (2023) |
| AB1532 | 48 | Lecce | Vernole | Italy | Belluardo *et al.* (2023) |
| AB1537 | 48 | Lecce | Vernole | Italy | Belluardo *et al.* (2023) |
| AB1538 | 48 | Lecce | Vernole | Italy | Belluardo *et al.* (2023) |
| AB1539 | 48 | Lecce | Vernole | Italy | Belluardo *et al.* (2023) |
| AB1540 | 48 | Lecce | Vernole | Italy | Belluardo *et al.* (2023) |
| AB1541 | 48 | Lecce | Vernole | Italy | Belluardo *et al.* (2023) |
| AB1542 | 48 | Lecce | Vernole | Italy | Belluardo *et al.* (2023) |
| AB1543 | 48 | Lecce | Vernole | Italy | Belluardo *et al.* (2023) |
| AB2472 | 49 | Cosenza | San Lucido | Italy | Belluardo *et al.* (2023) |
| AB2485 | 49 | Cosenza | San Lucido | Italy | Belluardo *et al.* (2023) |
| AB2487 | 49 | Cosenza | San Lucido | Italy | Belluardo *et al.* (2023) |
| AB2488 | 49 | Cosenza | San Lucido | Italy | Belluardo *et al.* (2023) |
| AB2489 | 49 | Cosenza | San Lucido | Italy | Belluardo *et al.* (2023) |
| AB2491 | 49 | Cosenza | San Lucido | Italy | Belluardo *et al.* (2023) |
| AB2501 | 49 | Cosenza | San Lucido | Italy | Belluardo *et al.* (2023) |
| AB2503 | 49 | Cosenza | San Lucido | Italy | Belluardo *et al.* (2023) |
| AB2504 | 49 | Cosenza | San Lucido | Italy | Belluardo *et al.* (2023) |
| AB2510 | 49 | Cosenza | San Lucido | Italy | Belluardo *et al.* (2023) |

* Refers to Harris, et al. ^2,^Harris, et al. ^3,^Perera and Harris ^4,^Rato, et al. ^5,^Rato, et al. ^6^.

** Samples collected with the permit SF/0213/21 (Generalitat de Catalunya) for the individuals from Barcelona; Prefectural Decree number 2017-67A for the samples collected in continental France; and Prefectural Decree number 2A-2020-07-22-002 and 2B-2020-07-29-002 for the samples collected in Corsica.

**Table S2.** Chi2 values obtained from the Hardy-Weinberg equilibrium test (HWE) and detection of null alleles for each locus. HWE significance values were corrected with the False Discovery rate test, where the adjusted *p* < 0.05 is identified as *, p < 0.001 as **, and *p* < 0.00e^-20^ as ***. Statistically significant values are in bold.

| **Locus** | **HWE** | **Null Present** |
| --- | --- | --- |
| Mt3 | **404.0923** *** | Yes |
| Mt6 | 97.3796 | No |
| Mt7 | **79.4751** ** | No |
| Mt11 | 35.3120 | No |
| Mt13 | **153.4888** ** | No |
| Mt14 | **271.2507** *** | Yes |
| Mt16 | 58.2705 | No |
| Mt21 | **125.9197** ** | No |
| Mt24 | **105.2326** * | No |
| Mt27 | **147.5621** ** | No |
| Mt29 | **212.2475** *** | No |

**Table S3.** Genetic diversity statistics calculated from the microsatellite markers for each of the 44 populations of *Tarentola* *mauritanica* used in this study. Population number matches the code from Table S1. Minimum and maximum values are highlighted in bold.

| **Population** | **N** | **N_a_** | **Ar** | **R-Ar** | **H_o_** | **H_e_** |
| --- | --- | --- | --- | --- | --- | --- |
| pop3 | 6 | 19 | 2.09 | 2.06 | 0.38 | 0.43 |
| pop4 | 7 | 16 | 1.83 | 1.71 | 0.27 | 0.29 |
| pop5 | 7 | 17 | 1.84 | 1.70 | 0.29 | 0.29 |
| pop6 | 5 | 19 | 2.17 | 2.02 | 0.40 | 0.39 |
| pop7 | 7 | 23 | 2.46 | 2.21 | 0.45 | 0.45 |
| pop8 | 6 | 25 | 2.58 | 2.49 | 0.49 | 0.55 |
| pop9 | 6 | 20 | 2.13 | 2.03 | 0.28 | 0.39 |
| pop12 | 26 | **56** | **3.83** | **2.97** | **0.68** | **0.70** |
| pop13 | 5 | 23 | 2.48 | 2.45 | 0.53 | 0.54 |
| pop14 | 5 | 29 | 3.05 | 2.76 | 0.55 | 0.57 |
| pop15 | 5 | 22 | 2.53 | 2.36 | 0.58 | 0.51 |
| pop16 | 5 | 27 | 2.91 | 2.60 | 0.50 | 0.52 |
| pop17 | 43 | 33 | 2.63 | 2.26 | 0.46 | 0.51 |
| pop18 | 10 | 21 | 2.29 | 2.12 | 0.45 | 0.51 |
| pop19 | 60 | 34 | 2.64 | 2.20 | 0.45 | 0.49 |
| pop20 | 10 | 24 | 2.44 | 2.18 | 0.59 | 0.50 |
| pop21 | 10 | 25 | 2.35 | 2.04 | 0.39 | 0.39 |
| pop22 | 33 | 35 | 2.84 | 2.35 | 0.49 | 0.52 |
| pop23 | 48 | 30 | 2.36 | 2.00 | 0.45 | 0.42 |
| pop24 | 8 | 21 | 2.24 | 2.14 | 0.33 | 0.44 |
| pop25 | 7 | 21 | 2.22 | 1.99 | 0.36 | 0.39 |
| pop27 | 8 | 17 | 1.80 | 1.75 | **0.16** | 0.32 |
| pop28 | 5 | 18 | 2.02 | 2.02 | 0.48 | 0.42 |
| pop29 | 8 | 21 | 2.28 | 2.04 | 0.45 | 0.42 |
| pop30 | 9 | 23 | 2.28 | 2.06 | 0.32 | 0.42 |
| pop31 | 10 | 17 | 1.84 | 1.68 | 0.29 | 0.29 |
| pop32 | 9 | 15 | **1.57** | **1.43** | 0.18 | **0.18** |
| pop33 | 7 | 17 | 1.85 | 1.75 | 0.29 | 0.30 |
| pop34 | 10 | 17 | 1.87 | 1.69 | 0.31 | 0.29 |
| pop35 | 10 | **14** | 1.65 | 1.55 | 0.31 | 0.26 |
| pop36 | 10 | 22 | 2.34 | 2.05 | 0.41 | 0.42 |
| pop37 | 10 | 20 | 2.00 | 1.81 | 0.35 | 0.33 |
| pop38 | 10 | 26 | 2.60 | 2.24 | 0.44 | 0.48 |
| pop39 | 10 | 22 | 2.22 | 1.98 | 0.34 | 0.40 |
| pop40 | 6 | 20 | 2.29 | 2.11 | 0.46 | 0.44 |
| pop41 | 5 | 23 | 2.49 | 2.31 | 0.42 | 0.44 |
| pop42 | 14 | 17 | 1.94 | 1.77 | 0.32 | 0.32 |
| pop43 | 17 | 16 | 1.75 | 1.69 | 0.29 | 0.32 |
| pop44 | 10 | 18 | 1.94 | 1.85 | 0.31 | 0.38 |
| pop45 | 20 | 23 | 2.21 | 1.96 | 0.38 | 0.39 |
| pop46 | 12 | 18 | 1.84 | 1.67 | 0.23 | 0.29 |
| pop47 | 10 | 19 | 2.14 | 1.91 | 0.44 | 0.38 |
| pop48 | 17 | 17 | 2.06 | 1.94 | 0.44 | 0.44 |
| pop49 | 10 | 17 | 1.90 | 1.79 | 0.36 | 0.35 |

The following parameters are displayed: sample size (N), mean number of alleles (N_a_), allelic richness (Ar), rarefied allelic richness (R-Ar), observed heterozygosity (H_o_) and expected heterozygosity (H_e_).

**Table S4.** G’’_st_ pairwise statistical analysis among all defined geographic regions. Numbers in bold denote the ones significantly different from zero (95% CI).

| **Populations** | **Corsica** | **France** | **Greece** | **Morocco** | **Iberia** | **Balearic Islands** | **Tunisia** | **Madeira** |
| --- | --- | --- | --- | --- | --- | --- | --- | --- |
| Corsica |  |  |  |  |  |  |  |  |
| France | **0.0745** |  |  |  |  |  |  |  |
| Greece | 0.1177 | **0.2222** |  |  |  |  |  |  |
| Morocco | **0.528** | **0.4633** | **0.5529** |  |  |  |  |  |
| Iberia | **0.2402** | **0.2704** | **0.242** | **0.3145** |  |  |  |  |
| Balearic Islands | **0.1558** | **0.1984** | **0.2646** | **0.4799** | **0.177** |  |  |  |
| Tunisia | **0.1471** | **0.2512** | **0.149** | **0.5323** | **0.268** | **0.2807** |  |  |
| Madeira | **0.207** | **0.2111** | **0.2387** | **0.497** | **0.263** | **0.2594** | **0.2902** |  |
| Italy | 0.0422 | **0.0767** | **0.1902** | **0.4835** | **0.217** | **0.1117** | 0.1567 | **0.1828** |

**Table S5.** F_st_ pairwise statistical analysis among all defined geographic regions. Numbers in bold denote the ones significantly different from zero (95% CI).

| **Populations** | **Corsica** | **France** | **Greece** | **Morocco** | **Iberia** | **Balearic Islands** | **Tunisia** | **Madeira** |
| --- | --- | --- | --- | --- | --- | --- | --- | --- |
| **Corsica** |  |  |  |  |  |  |  |  |
| **France** | **0.0363** |  |  |  |  |  |  |  |
| **Greece** | 0.053 | **0.1081** |  |  |  |  |  |  |
| **Morocco** | **0.189** | **0.1766** | **0.1692** |  |  |  |  |  |
| **Iberia** | **0.1118** | **0.1287** | **0.1048** | **0.1197** |  |  |  |  |
| **Balearic Islands** | **0.0837** | **0.1038** | **0.1351** | **0.1679** | **0.0807** |  |  |  |
| **Tunisia** | **0.0756** | **0.1299** | **0.0633** | **0.1748** | **0.1233** | **0.1479** |  |  |
| **Madeira** | **0.1061** | **0.1092** | **0.1128** | **0.1862** | **0.1242** | **0.1333** | **0.1463** |  |
| **Italy** | 0.0189 | **0.0398** | **0.0921** | **0.2007** | **0.1052** | **0.0566** | **0.0811** | **0.096** |

**Table S6.** Historical gene flow with 95% confident intervals estimates among the major geographic groups of *Tarentola mauritanica* using MIGRATE. Source populations = rows, sink populations = columns.

| **Pop_ID** | **Corsica** | **France** | **Greece** | **Morocco** | **Iberia** | **Balearic** | **Tunisia** | **Madeira** | **Italy** |
| --- | --- | --- | --- | --- | --- | --- | --- | --- | --- |
| **Corsica** |  | 35.397  (14.667-56) | 28.155  (8-47.333) | 22.932  (3.333-42) | 26.514  (6.667-46) | 27.34  (7.333-46) | 27.557  (1.333-60) | 25.992  (6.667-44.667) | 40.804  (20-61.333) |
| **France** | 35.88  (12.667-58.667) |  | 34.031  (12-56) | 46.472  (22-70.667) | 41.218  (20.667-61.333) | 37.322  (0-37.333) | 25.804  (4.667-46.667) | 70.28  (49.333-90.667) | 78.68  (56.667-98.667) |
| **Greece** | 16.324  (0-32.667) | 13.669  (0-30.667) |  | 17.277  (0-34) | 18.567  (0.667-36) | 21.569  (2-40) | 21.764  (0-32) | 29.548  (8.667-50) | 34.662  (14.667-54) |
| **Morocco** | 32.493  (6.667-64.667) | 31.317  (8-53.333) | 47.5  (24-70) |  | 38.615  (18.667-57.333) | 60.402  (33.333-86.667) | 33.652  (8-59.333) | 41.193  (21.333-60.667) | 35.336  (15.333-54.667) |
| **Iberia** | 39.957  (19.333-60) | 85.993  (62-109.333) | 41.298  (20-62) | 96.138  (51.333-92) |  | 78.104  (55.333-101.333) | 87.668  (60.667-113.333) | 105.06  (79.333-130.667) | 124.274  (99.333-148) |
| **Balearic** | 50.825  (22-78) | 55.671  (34.667-76.667) | 29.335  (8.667-48.667) | 36.211  (13.333-58.667) | 18.589  (0.667-36) |  | 31.528  (9.333-53.333) | 32.246  (11.333-52) | 30.632  (8-52.667) |
| **Tunisia** | 41.07  (18.667-62.667) | 37.141  (16.667-57.333) | 33.215  (5.333-58) | 34.193  (14-54) | 22.397  (4-40) | 20.288  (1.333-38) |  | 30.297  (2.667-54.667) | 29.427  (10-48.667) |
| **Madeira** | 27.773  (7.333-46.667) | 42.435  (21.333-62.667) | 39.108  (11.333-64) | 39.55  (15.333-62.667) | 59.676  (40-78.667) | 41.977  (21.333-61.333) | 62.701  (38-87.333) |  | 51.166  (30.667-71.333) |
| **Italy** | 45.361  (24-66) | 68.695  (34.667-103.333) | 22.444  (0-48) | 59.334  (33.333-84.667) | 80.438  (54-110.667) | 31.536  (10.667-52) | 54.858  (32.667-76.667) | 109.946  (74-128) |  |

**Table S7.** Contemporary gene flow with 95% confident intervals estimates among the major geographic groups of *Tarentola mauritanica* using Bayesass. Source populations = rows, sink populations = columns.

| **Pop_ID** | **Corsica** | **France** | **Greece** | **Morocco** | **Iberia** | **Balearic** | **Tunisia** | **Madeira** | **Italy** |
| --- | --- | --- | --- | --- | --- | --- | --- | --- | --- |
| **Corsica** | 0.6776  (0.6566-0.6986) | 0.0056  (-0.0056-0.0168) | 0.0223  (-0.0177-0.0623) | 0.0506  (-0.0110-0.1121) | 0.0015  (-0.0014-0.0044) | 0.0122  (-0.0111-0.0355) | 0.0122  (-0.0107-0.0351) | 0.0056  (-0.0038-0.0150) | 0.0026  (-0.0021-0.0073) |
| **France** | 0.0141  (-0.0159-0.0441) | 0.7305  (0.5913-0.8697) | 0.025  (-0.0205-0.0705) | 0.011  (-0.0123-0.0343) | 0.0019  (-0.0016-0.0054) | 0.0135  (-0.0112-0.0382) | 0.015  (-0.0132-0.0432) | 0.0091  (-0.0083-0.0265) | 0.0056  (-0.0089-0.0201) |
| **Greece** | 0.0106  (-0.0102-0.0314) | 0.0056  (-0.0050-0.0162) | 0.6883  (0.6481-0.7285) | 0.0059  (-0.0055-0.0173) | 0.0015  (-0.0016-0.0046) | 0.012  (-0.0103-0.0343) | 0.0122  (-0.0109-0.0353) | 0.005  (-0.0048-0.0148) | 0.0029  (-0.0033-0.0092) |
| **Morocco** | 0.0112  (-0.0098-0.0322) | 0.0071  (-0.0054-0.0196) | 0.0228  (-0.0186-0.0642) | 0.84  (0.7675-0.9125) | 0.0016  (-0.0019-0.0051) | 0.0121  (-0.0099-0.0341) | 0.0126  (-0.0105-0.0357) | 0.005  (-0.0050-0.0150) | 0.0034  (-0.0035-0.0106) |
| **Iberia** | 0.0174  (-0.0104-0.0452) | 0.04  (0.0043-0.0757) | 0.0392  (-0.0149-0.0933) | 0.056  2(0.0143-0.0982) | 0.9582  (0.9382-0.9782) | 0.123  5(0.0557-0.1913) | 0.0174  (-0.0140-0.0488) | 0.0262  (-0.0034-0.0558) | 0.0049  (-0.0045-0.0143) |
| **Balearic** | 0.0108  (-0.0094-0.0310) | 0.0058  (-0.0056-0.01717) | 0.0224  (-0.0190-0.0638) | 0.0059  (-0.0057-0.0175) | 0.001  (-0.0010-0.0030) | 0.6787  (0.6562-0.7012) | 0.0128  (-0.0115-0.0371) | 0.0053  (-0.0041-0.0147) | 0.0027  (-0.0026-0.0080) |
| **Tunisia** | 0.0107  (-0.0099-0.0313) | 0.0057  (-0.0049-0.0163) | 0.0223  (-0.0191-0.0637) | 0.0058  (-0.0056-0.0172) | 0.0015  (-0.0012-0.0042) | 0.0121  (-0.0110-0.0352) | 0.679  (0.6555-0.7025) | 0.0049  (-0.0053-0.0151) | 0.0028  (-0.0023-0.0079) |
| **Madeira** | 0.0134  (-0.0107-0.0375) | 0.0124  (-0.0092-0.0340) | 0.0295  (-0.0223-0.0813) | 0.0172  (-0.0104-0.0448) | 0.0056  (-0.0032-0.0144) | 0.0565  (0.0014-0.1116) | 0.0257  (-0.0141-0.0655) | 0.9053  (0.8579-0.9527) | 0.0039  (-0.0039-0.0117) |
| **Italy** | 0.2342  (0.1764-0.2920) | 0.1873  (0.0405-0.3341) | 0.1282  (0.0448-0.2117) | 0.0074  (-0.0069-0.0217) | 0.0272  (0.00917-0.0452) | 0.0793  (0.0082-0.1505) | 0.213  (0.1466-0.2794) | 0.0337  (-0.0047-0.0721) | 0.9713  (0.9490-0.9936) |

**FIGURES**

**Figure S1.** Delta K estimation according to Evanno *et al* ^7^’s method.


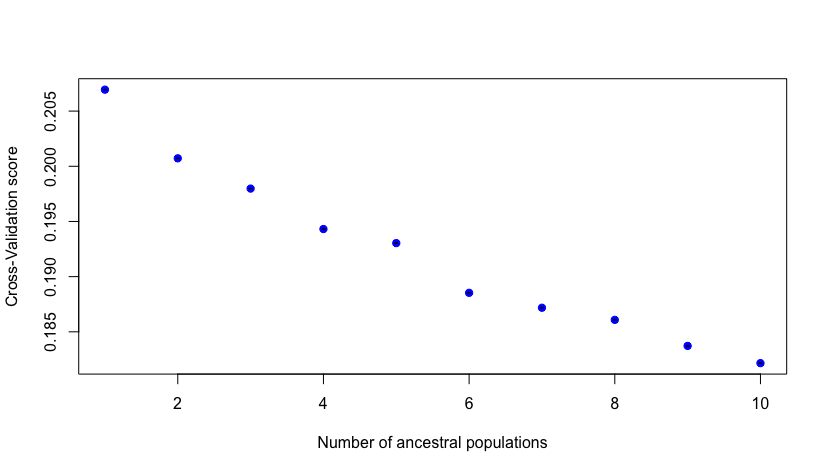


**Figure S2.** Cross-validation score from tess3r plotted against the number of ancestral populations (*K*) of Moorish gecko. Cross-validation is performed through calculating root mean-squared errors for a subset of loci. Usually, the best *K* is found where the cross-validation score starts to plateau.


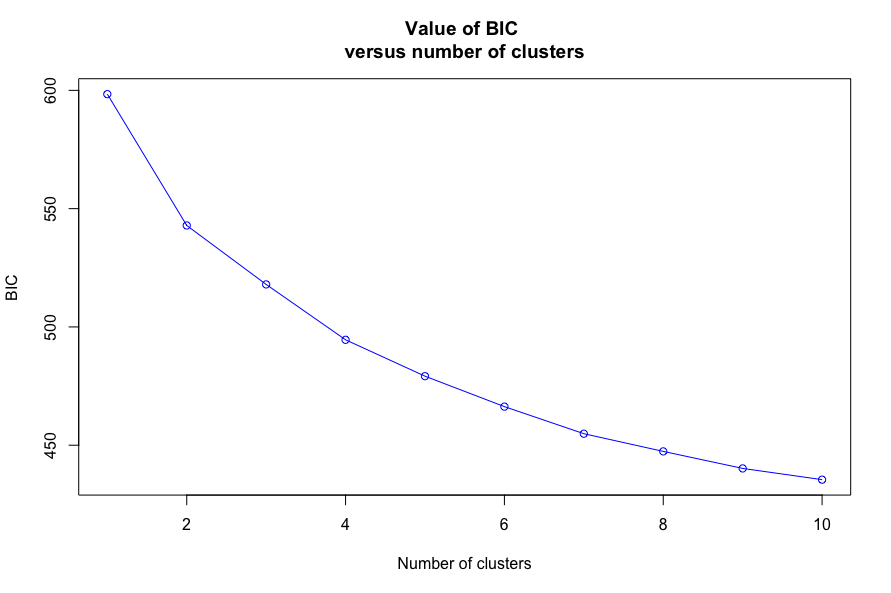


**Figure S3.** Graph of the Bayesian Information Criterion (BIC) values for increasing values of K, during the Discriminant of Principal Components (DAPC) analysis. In practice, the “best” BIC is often indicated by an elbow in the curve of BIC values as a function of K.


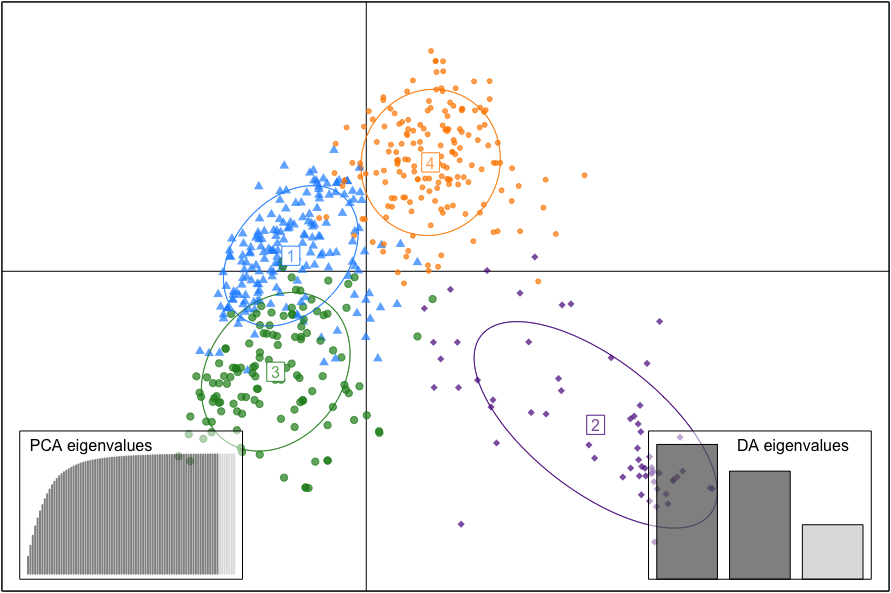


**Figure S4.** Discriminant analysis of Principal Components (DAPC) of all populations of *T. mauritanica*. Colours match with STRUCTURE genetic clusters.

**REFERENCES**

1 Belluardo, F. *et al.* Multilocus genetic assessment of the *Tarentola* *mauritanica* clade III in the Italian Peninsula and main islands: new insights into the evolutionary history of the clade. *Journal of Zoological Systematics and Evolutionary Research* **submitted** (2023).

2 Harris, D. J., Batista, V., Carretero, M. A. & Ferrand, N. Genetic variation in *Tarentola* *mauritanica* (Reptilia: Gekkonidae) across the Strait of Gibraltar derived from mitochondrial and nuclear DNA sequences. *Amphibia-Reptilia* **25**, 451-459 (2004).

3 Harris, D. J., Batista, V., Lymberakis, P. & Carretero, M. A. Complex estimates of evolutionary relationships in *Tarentola* *mauritanica* (Reptilia: Gekkonidae) derived from mitochondrial DNA sequences. *Molecular Phylogenetics and Evolution* **30**, 855-859 (2004).

4 Perera, A. & Harris, D. J. Genetic diversity in the gecko *Tarentola* *mauritanica* within the Iberian Peninsula. *Amphibia-Reptilia* **29**, 583-588 (2008).

5 Rato, C., Carranza, S. & Harris, D. J. Evolutionary history of the genus *Tarentola* (Gekkota: Phyllodactylidae) from the Mediterranean Basin, estimated using multilocus sequence data. *BMC Evolutionary Biology* **12**, 10.1186/1471-2148-1112-1114 (2012).

6 Rato, C., Carranza, S., Perera, A., Carretero, M. A. & Harris, D. J. Conflicting patterns of nucleotide diversity between mtDNA and nDNA in the Moorish gecko, *Tarentola* *mauritanica*. *Molecular Phylogenetics and Evolution* **56**, 962-971 (2010).

7 Evanno, G., Regnaut, S. & Goudet, J. Detecting the number of clusters of individuals using the software STRUCTURE: a simulation study. *Molecular Ecology* **14**, 2611-2620 (2005).
